# Cannabigerol and Cannabichromene Induce Lung Cancer Cell Death and Apoptosis—Contribution of PPARα to Cannabigerol Effects

**DOI:** 10.3390/antiox15060754

**Published:** 2026-06-15

**Authors:** Theresa Spengler, Felix Wittig, Marcus Frank, Burkhard Hinz

**Affiliations:** 1 Institute of Pharmacology and Toxicology, Rostock University Medical Center, 18057 Rostock, Germany; 2 Electron Microscopy Center, Rostock University Medical Center, 18057 Rostock, Germany; 3 Department Life, Light and Matter, University of Rostock, 18059 Rostock, Germany

**Keywords:** cannabigerol, cannabichromene, lung cancer, peroxisome proliferator-activated receptor α, cell death, apoptosis, mitochondrial dysfunction

## Abstract

Cannabinoids are potential anticancer agents for the add-on treatment of malignant tumors. Here, the effects of the previously less-explored non-psychoactive phytocannabinoids cannabigerol (CBG) and cannabichromene (CBC) on survival, apoptosis, and mitochondrial function were assessed in A549 and H460 lung cancer cells. CBG and CBC triggered concentration-dependent cell death, autophagy, and mitochondrial apoptosis in both cell lines, with apoptosis indicated by Annexin V staining, activation of caspase-8, -9, and -3/7, loss of mitochondrial membrane potential, and elevated cytosolic levels of mitochondrial cytochrome c. CBG also upregulated ATF4, a stress-responsive transcription factor involved in autophagy and apoptotic signaling, and enhanced PARP cleavage. Both cannabinoids increased mitochondrial superoxide formation and reduced the mitochondrial oxygen consumption rate, with CBG additionally decreasing NDUFB8, a subunit of respiratory chain complex I. Pharmacological receptor modulation showed that CBG- and CBC-induced cell death occurred independently of CB_1_, CB_2_, TRPV1, TRPM8, and PPARγ, whereas CBG-mediated cell death relied on PPARα, which also contributed to its apoptotic effects. In summary, CBG and CBC induce apoptosis and cell death in A549 and H460 cells, with PPARα mediating the effects of CBG, highlighting its potential as a therapeutic target.

## 1. Introduction

There is now substantial preclinical evidence supporting an anticancer action of various cannabinoids in different tumor entities. This effect extends to inhibition of tumor cell proliferation, angiogenesis, invasion and metastasis, as well as induction of apoptosis and autophagy (for reviews, see [1,2]). In addition, synergy between cannabinoids and established chemotherapeutics at the level of tumor cell proliferation and apoptosis was demonstrated in many cases (for review, see [3]). In accordance with this, a first randomized phase Ib clinical study showed that adding nabiximols, an extract containing roughly equal amounts of Δ^9^-tetrahydrocannabinol (THC) and cannabidiol (CBD), to existing chemotherapy resulted in an increase in survival time in patients with recurrent glioblastoma [4].

Two of the less well-researched phytocannabinoids, in comparison to THC and CBD, are the non-psychoactive representatives cannabigerol (CBG) and cannabichromene (CBC). The monocyclic CBG was first isolated by Gaoni and Mechoulam in 1964 [5] and was considered the missing link in the biosynthesis of THC. In 1971, it was synthesized de novo by its discoverers [6]. CBC, a structurally diverse bicyclic cannabinoid, was identified in 1966 [7] and, along with THC, is one of the most abundant phytocannabinoids. CBG and CBC interact with the endocannabinoid system in different ways. CBG has been shown to display a weak binding affinity to the cannabinoid receptors CB_1_ and CB_2_ [8,9], but to be the most potent phytocannabinoid ligand at the transient receptor potential vanilloid 1 (TRPV1) channel [10]. Furthermore, CBG is a potent antagonist of TRPM8 [11] and transcriptionally activates peroxisome proliferator-activated receptor (PPAR) α [12]. CBC also displays CB_1_ and CB_2_ affinities [13], exhibits selectivity and high efficacy at the CB_2_ receptor [14], and activates TRPA1 [11].

As early as 2006, Ligresti et al. reported in vitro anticancer effects of CBG and CBC [15]. However, despite corresponding efforts for CBG (for review, see [16]), the mechanistic basis of the tumor cell death-inducing effect of both cannabinoids was less investigated than that of other phytocannabinoids such as CBD. In particular, the PPARα-activating property of CBG has not been considered in the context of cancer so far. Remarkably, several findings published in recent years have demonstrated that activation of the transcription factor PPARα in cancer cells is associated with antiproliferative [17], cytotoxic [18,19], pro-apoptotic [20], and mitochondrial dysfunction-promoting [20,21] effects. On the other hand, several studies have also shown that antagonists of PPARα possess an antiproliferative, viability-reducing or pro-apoptotic action on tumor cells [22,23,24,25]. In fact, due to their relationship with tumor metabolism, the genes regulated by PPARα can mediate cancer-promoting or cancer-inhibiting effects (for review, see [26]), making PPARα a complex target and offering therapeutic potential for both agonists and antagonists of this receptor.

This study investigates the effects of CBG and CBC on lung cancer cell survival, apoptosis, and mitochondrial function and bioenergetics, with particular emphasis on the role of PPARα in this process. Here, we show convincing cytotoxic, pro-apoptotic and mitochondrial toxic effects of both cannabinoids. More importantly, this study demonstrates for the first time a mediating role of PPARα in the induction of tumor cell death and apoptosis by CBG, which makes this non-psychoactive phytocannabinoid an interesting compound in the search for new targeted therapies for the treatment of malignant tumors.

## 2. Materials and Methods

### 2.1. Materials

Cannabigerol (CBG, #15293), (±)-cannabichromene (CBC, #ISO60163), AM251 (#71670), AM630 (#10006974), GW6471 (#11697), and GW9662 (#70785) were obtained from Cayman Chemical (Ann Arbor, MI, USA). Capsazepine (#C191) was obtained from Sigma-Aldrich (Taufkirchen, Germany). Icilin (#1531) and WS12 (#3040) were purchased from Tocris (Bristol, UK). Etomoxir (#HY-50202; MedChem Express, Monmouth Junction, NJ, USA) and NXT629 (#HY-114263; MedChem Express, Monmouth Junction, NJ, USA) were obtained from Hölzel Diagnostika Handels GmbH (Cologne, Germany). Leupeptin was bought from Biomol (Hamburg, Germany). Acetic acid, dimethyl sulfoxide (DMSO), ethylenediaminetetraacetic acid (EDTA), glycerol, hydrochloric acid (HCl, 37%), sodium chloride (NaCl), sodium hydroxide (NaOH), and Tris hydrochloride (Tris-HCl) were obtained from AppliChem (Darmstadt, Germany). Aprotinin, bromophenol blue, hydrogen peroxide solution (H_2_O_2_, 30%), luminol, orthovanadate, *p*-coumaric acid, thiazolyl blue tetrazolium bromide (MTT), and phenylmethanesulfonyl fluoride (PMSF) were obtained from Sigma-Aldrich (Taufkirchen, Germany). Methanol was purchased from J. T. Baker (Griesheim, Germany), ethanol from Walter-CMP (Kiel, Germany), and aqua ad iniectabilia from B. Braun Melsungen (Melsungen, Germany). 4-(2-hydroxyethyl)-1-piperazineethanesulfonic acid (HEPES) and β-mercaptoethanol were obtained from Ferak Berlin (Berlin, Germany). Acrylamide (Rotiphorese^®^ Gel, 30%), ammonium peroxydisulphate (APS), crystal violet, glycine, non-fat milk (NFM), Ponceau S, sodium dodecyl sulfate (SDS) ultrapure, Tris ultrapure, N,N,N′,N′-tetramethylethylenediamine (TEMED), Triton^®^ X-100, and Tween^®^ 20 were purchased from Carl Roth (Karlsruhe, Germany). Bovine serum albumin (BSA) was obtained from SERVA (Heidelberg, Germany). Dulbecco’s phosphate-buffered saline (DPBS, #P04-36500), fetal bovine serum (FBS, #P30-3306) and High Glucose Dulbecco’s Modified Eagle Medium (DMEM, #P04-04510) were obtained from PAN-Biotech (Aidenbach, Germany). Penicillin-streptomycin (#15140-122) and 0.5% Trypsin-EDTA (#15400-054) were purchased from Thermo Fisher Scientific (Schwerte, Germany).

### 2.2. Cell Culture

A549 cells (#ACC-107; RRID:CVCL_0023) were obtained from the German Collection of Microorganisms and Cell Cultures (DSMZ, Braunschweig, Germany), while NCI-H460 cells (#HTB-177, RRID:CVCL_0459) were purchased from the American Type Culture Collection (ATCC, Manassas, VA, USA). Both cell lines were cultured in DMEM supplemented with 10% (*v*/*v*) heat-inactivated FBS, 100 U/mL penicillin, and 100 µg/mL streptomycin (hereafter referred to as serum-containing DMEM) in a humidified incubator at 37 °C and 5% CO_2_. Cells between passages 2 and 20 were used throughout the study. Seeding was performed at a density of approximately 4.4 × 10^4^ cells/cm^2^, except for colony formation assays and Seahorse experiments.

Incubation with test substances or their vehicles was performed in serum-free DMEM supplemented with 100 U/mL penicillin and 100 μg/mL streptomycin (hereafter referred to as serum-free DMEM) after washing the cells with DPBS. The test substances were dissolved in DMSO (CBG, CBC, AM251, AM630, capsazepine, etomoxir, GW6471, GW9662, icilin, NXT629) or ethanol (WS12). The final concentration of solvents in the incubation media of cells treated with a test substance and its vehicle did not exceed 0.1% (*v*/*v*) for ethanol or DMSO, with a maximum of 0.3% (*v*/*v*) DMSO in settings involving simultaneous incubation with a cannabinoid and two antagonists.

### 2.3. Cell Survival and Metabolic Activity Assays

A549 and H460 cells were plated in 96-well plates at a density of 1.5 × 10^4^ cells per well in serum-containing DMEM and allowed to adhere for 24 h. Subsequently, cells were exposed to the indicated compounds in serum-free DMEM for the respective incubation periods, after which cell viability and metabolic activity were evaluated.

Cell survival was determined by crystal violet staining of adherent viable cells [27]. Cells were fixed overnight with ice-cold absolute ethanol and subsequently stained with 100 µL of crystal violet solution (0.1% (*w*/*v*) in 10% (*v*/*v*) ethanol) for 30 min under light-protected conditions. Excess dye was removed by washing and the remaining dye was dissolved in 10% (*v*/*v*) acetic acid. Absorbance was measured at 570 nm using a microplate reader (Infinite F200 Pro, Tecan, Männedorf, Switzerland).

Cellular metabolic activity was determined using MTT, a tetrazolium dye that is reduced by metabolically active cells to insoluble formazan crystals. Cells were incubated with MTT (final concentration of 0.5 mg/mL) for 2 h at 37 °C and 5% CO_2_, protected from light. The media were then removed, and 100 µL of 100% DMSO was added for 1 h to dissolve the formazan completely. Measurements of absorbance were performed at 570 nm, with a 690 nm reference, using an Infinite F200 Pro microplate reader.

### 2.4. Colony Formation Assay

In 6-well plates, 500 cells per well were seeded in serum-containing DMEM and incubated for 48 h to allow cell attachment and early proliferation prior to treatment. Thereafter, the medium was replaced with fresh serum-containing DMEM containing CBG, CBC, or vehicle control, and cells were incubated for 7 days to assess effects on colony formation. Subsequently, cells were fixed in ice-cold absolute ethanol (−20 °C) and stained with 0.1% (*w*/*v*) crystal violet. Images were captured with a digital camera, and colonies were quantified using the open-source software Fiji (ImageJ) version 1.54f.

### 2.5. Annexin V/7-AAD Binding Assay

Cells were seeded in 12-well plates at 1.65 × 10^5^ cells per well and allowed to attach for 24 h in serum-containing DMEM, before being treated for 6 or 24 h with the test substances or their vehicle in serum-free DMEM. After incubation, cells were detached using trypsin-EDTA, collected together with the medium, and centrifuged at 200× *g* and 4 °C for 5 min. The resulting cell pellet was resuspended in ice-cold DPBS. The proportions of live cells, early and late apoptotic cells, and dead cells were quantified using the Muse^®^ Annexin V & Dead Cell Kit (#MCH100105). Staining with Annexin V and 7-AAD was performed according to the manufacturer’s protocol, and data were acquired using the Cytek^®^ Guava^®^ Muse^®^ cell analyzer (Cytek Biosciences, Amsterdam, The Netherlands).

### 2.6. Cell Cycle Assay

Cells were seeded in 6-cm Petri dishes at 9.4 × 10^5^ cells per dish and allowed to attach for 24 h in serum-containing DMEM, followed by treatment with the cannabinoids or their vehicle for 24 h in serum-free DMEM. After incubation, cells were harvested with trypsin-EDTA. Cells and their supernatants were centrifuged at 300× *g* and 4 °C for 5 min. The resulting cell pellet was washed with DPBS and centrifuged again at 300× *g* and 4 °C for 5 min. Subsequently, the cell pellet was resuspended in 50 µL DPBS and carefully fixed with 1 mL of ice-cold 70% ethanol. Cells were stored at −20 °C until analysis. For cell cycle measurement, cells were processed according to the kit manufacturer’s instructions provided with the Muse^®^ Cell Cycle Kit (#MCH100106). The percentages of cells in the G0/G1, S, and G2/M phases of the cell cycle were determined with the Cytek^®^ Guava^®^ Muse^®^ cell analyzer (Cytek Biosciences).

### 2.7. Caspase Activity Assays

A549 and H460 cells were plated in 96-well plates at a density of 1.5 × 10^4^ cells per well and maintained in serum-containing DMEM for 24 h. Subsequently, cells were treated with the respective test compounds in serum-free DMEM for the indicated durations. Promega luciferase assays were used to measure caspase activity (Caspase-Glo^®^ 3/7 assay, #G8091; Caspase-Glo^®^ 8 assay, #G8201; Caspase-Glo^®^ 9 assay, #G8211). For this purpose, the corresponding Caspase-Glo^®^ reagents were added to the wells in accordance with the manufacturer’s instructions and incubated in the dark at room temperature for 1 h. The luminescence was detected using a microplate reader (Infinite F200 Pro).

### 2.8. Detection of Mitochondrial Superoxide

A549 and H460 cells were plated in 96-well plates at a density of 1.5 × 10^4^ cells per well and maintained in serum-containing DMEM for 24 h. Cells were then treated with the respective test compound or its vehicle in serum-free DMEM and incubated for 2 h or 6 h. After the incubation period, nuclei were stained with Hoechst dye (1 µg/mL final concentration; Merck, Darmstadt, Germany) by adding the staining solution to each well and incubating the cells for 30 min at 37 °C and 5% CO_2_, protected from light. Mitochondrial superoxide was then detected using MitoSOX™ Red (#M36008, Thermo Fisher Scientific; 5 µM final concentration in serum-free DMEM), with an additional 10 min incubation at 37 °C and 5% CO_2_, also protected from light. After removing the staining solution, 100 µL DPBS was added to each well, and fluorescence was measured using a microplate reader (Infinite F200 Pro; excitation/emission wavelengths: 360/465 nm for Hoechst and 535/595 nm for MitoSOX™ Red). Data were blank-corrected using wells containing cells without Hoechst and MitoSOX™ Red, and mitochondrial superoxide levels were quantified by calculating the ratio of MitoSOX™ Red to Hoechst fluorescence to account for differences in cell number. Finally, the values were normalized to the respective vehicle control.

### 2.9. Determination of Mitochondrial Membrane Potential

To assess changes in mitochondrial membrane potential, the JC-10 Mitochondrial Membrane Potential Assay Kit (#22204, AAT Bioquest, Pleasanton, CA, USA) was used. For this purpose, A549 and H460 cells were seeded in 96-well plates at a density of 1.5 × 10^4^ cells per well and cultured for 24 h in serum-containing DMEM, followed by treatment with the respective compounds in serum-free DMEM. After 2 and 6 h of incubation, the cells were washed with 100 µL DPBS. Thereafter, 100 µL of a freshly prepared and pre-warmed JC-10 working solution (10 µM final concentration) in serum-free DMEM was added to the cells, and the plate was incubated at 37 °C and 5% CO_2_ for 45 min, protected from light. Finally, 100 µL DPBS was added to each well for measurement. Fluorescence was measured using a microplate reader with excitation/emission wavelengths of 485/535 nm (green fluorescence indicating depolarization) and 535/595 nm (orange fluorescence indicating healthy mitochondria). Blank correction was performed using wells containing cells without JC-10. Subsequently, data were analyzed by calculating the ratio of fluorescence intensities at emission wavelengths of 595 nm and 535 nm.

### 2.10. Seahorse XFe Analysis

Oxygen consumption rate (OCR) was determined using the Seahorse XFe24 Analyzer (Agilent Technologies, Waldbronn, Germany). A549 and H460 cells were seeded on Seahorse XF24 plates (Agilent Technologies) at a density of 2.5 × 10^4^ cells per well and cultured in serum-containing DMEM for 24 h. After washing with DPBS, cells were incubated with the indicated substances or vehicle in serum-free DMEM for 24 h. Prior to Seahorse XFe analysis, the medium was changed to Seahorse XF medium, pH 7.4 (#103575-100, Agilent Technologies), supplemented with 10 mM glucose, 2 mM glutamine and 1 mM pyruvate. Following medium exchange, cells were equilibrated in Seahorse XF medium at 37 °C for 1 h prior to OCR measurements. OCR was measured using the Seahorse XF Cell Mito Stress Test Kit (#103015-100, Agilent Technologies) in accordance with the manufacturer’s instructions and cell line–specific preliminary tests. Basal OCR was recorded, followed by sequential injection of oligomycin (1.5 μM; ATP synthase inhibitor; port A), carbonyl cyanide 4-(trifluoromethoxy)phenylhydrazone (FCCP, 2 μM for A549 or 1.5 μM for H460; uncoupling agent that dissipates the proton gradient and mitochondrial membrane potential; port B), and rotenone (complex I inhibitor) plus antimycin A (complex III inhibitor; 0.5 μM each; port C). Basal OCR and OCR after each injection were measured over three consecutive cycles per well. OCR values were normalized to protein content determined post-experiment. Therefore, 10 µL of lysis buffer (50 mM HEPES, 150 mM NaCl, 1 mM EDTA, 1% (*v*/*v*) Triton^®^ X-100, 10% (*v*/*v*) glycerol, 10 µg/mL aprotinin, 1 µg/mL leupeptin, 1 mM orthovanadate and 1 mM PMSF) was added to each well and the lysates were collected. Protein concentration was determined using the Pierce™ BCA Protein Assay Kit (#23225, Thermo Fisher Scientific).

The Seahorse XF Cell Mito Stress Test Report Generator (Agilent Technologies) was used to calculate basal respiration, ATP-linked respiration, spare respiratory capacity, and proton leak. Non-mitochondrial respiration, which represents the minimal respiration remaining after the injection of rotenone/antimycin A, was automatically subtracted from the values for basal respiration, spare respiratory capacity, and proton leak.

### 2.11. Total Cellular Protein Isolation

A549 or H460 cells were seeded in 10-cm dishes at a density of 2.5 × 10^6^ cells per dish and cultured for 24 h in serum-containing DMEM before treatment with compounds in serum-free DMEM. At the end of the incubation, supernatants were collected, cells were washed with DPBS, and detached using trypsin-EDTA. Supernatants, DPBS washes, and detached cells from each treatment group were combined and pelleted by centrifugation (200× *g*, 4 °C, 5 min). The cell pellet was washed with DPBS and centrifuged again under the same conditions. The resulting pellet was resuspended in lysis buffer (50 mM HEPES, 150 mM NaCl, 1 mM EDTA, 1% (*v*/*v*) Triton^®^ X-100, 10% (*v*/*v*) glycerol, 10 µg/mL aprotinin, 1 µg/mL leupeptin, 1 mM orthovanadate, 1 mM PMSF), incubated overnight at −20 °C, and centrifuged at 20,817× *g* and 4 °C for 5 min. The supernatant containing total cellular protein was collected and stored for subsequent protein analysis. Protein concentrations were determined using the Pierce™ BCA Protein Assay Kit.

### 2.12. Mitochondrial Protein Isolation

A549 and H460 cells were seeded, treated and harvested as in the whole-cell protein isolation experiments (Section 2.11). To achieve a higher total protein quantity, the detached cells of a treatment group from an independent experiment were combined from two Petri dishes each. After collecting the cell pellet by centrifugation (200× *g*, 4 °C, 5 min), it was washed in 0.9% NaCl and then centrifuged again (200× *g*, 4 °C, 5 min). Mitochondria and the cytosolic protein fraction were prepared using the Qproteome Mitochondria Isolation Kit (#37612, Qiagen, Hilden, Germany) according to the standard protocol provided by the manufacturer. Mitochondrial proteins were subsequently extracted by treating the mitochondrial fraction with the same lysis buffer and procedure used for total cellular protein isolation.

### 2.13. Western Blot Analysis

Equal amounts of protein were separated on a 10% (ATF4, CPT1A, PARP analysis) or 15% (all other proteins) SDS–polyacrylamide gel, transferred to a nitrocellulose membrane, and incubated for 1 h in 5% (*w*/*v*) NFM or BSA in Tris-buffered saline containing 0.1% (*v*/*v*) Tween^®^ 20 (TBS-T buffer). Following washing with TBS-T buffer, membranes were incubated overnight at 4 °C with primary antibodies in 1% (*w*/*v*) NFM (applies for β-actin, cytochrome c, LC3A/B, OxPhos antibodies, PARP) or 5% (*w*/*v)* BSA (applies for ATF4, cleaved PARP, CPT1A) in TBS-T buffer. The following antibodies were purchased from Cell Signaling Technology (Frankfurt am Main, Germany): ATF4 (#11815, RRID:AB_2616025), CPT1A (#97361, RRID:AB_2895298), cytochrome c (#11940, RRID:AB_2637071), LC3A/B (#4108, RRID:AB_2137703), PARP (#9532, RRID:AB_659884), and cleaved PARP (#32563S, RRID:AB_2799024). The OxPhos Human WB Antibody Cocktail (#45-8199, RRID:AB_2533836) was obtained from Thermo Fisher Scientific. β-Actin antibody (#A5441, RRID:AB_476744) was from Sigma-Aldrich. Antibody against VDAC1/Porin + VDAC3 (#ab14734, RRID:AB_443084) was bought from Abcam (Berlin, Germany).

After washing with TBS-T buffer, the membranes were incubated with horseradish peroxidase-conjugated secondary antibodies (anti-rabbit antibody, #7074; RRID:AB_2099233, or anti-mouse antibody, #7076, RRID:AB_330924; both from Cell Signaling Technology) in 1% (*w*/*v*) NFM in TBS-T buffer for 1 h at room temperature. Visualization was performed with a chemiluminescence solution containing 100 mM Tris-HCl (pH 8.5), 1.25 mM luminol, 200 µM *p*-coumaric acid, and 0.09% (*v*/*v*) H_2_O_2_, and antibody binding was detected using the ChemiDoc XRS gel documentation system (Bio-Rad Laboratories, Munich, Germany). Signal intensity was quantified using Quantity One 1-D Analysis Software Version 4.6.8 (Bio-Rad Laboratories). The intensity of each specific protein band was normalized to that of the loading control. As loading controls, the protein contents of β-actin (for total cell protein and proteins in cytosolic fractions) or VDAC (for proteins in mitochondrial fractions) were related to the respective target protein analyzed. For normalization against VDAC, the double band detectable with the VDAC1/Porin + VDAC3 antibody used was quantified, which is collectively referred to as VDAC in the corresponding blots.

Protein levels were expressed relative to the vehicle control. Band molecular weights were assigned using the Precision Plus Protein™ Dual Color Standard (Bio-Rad Laboratories). Following detection, membranes were stripped and reprobed for subsequent analysis.

### 2.14. Quantitative Real-Time Reverse Transcription Polymerase Chain Reaction (qRT-PCR)

Cells were seeded in 6-cm Petri dishes as described for the cell cycle assay (Section 2.6). Following incubation with test substances in serum-free DMEM, cells from each condition were collected using trypsin-EDTA, centrifuged at 200× *g* at 4 °C for 5 min, washed with DPBS, and centrifuged again under the same conditions. RLT buffer from the RNeasy Mini Kit (#74106, Qiagen, Hilden, Germany) was added to the cell pellet, and RNA isolation was performed according to the manufacturer’s instructions. RNA concentration was determined using a NanoDrop™ OneC microvolume UV-Vis spectrophotometer (Thermo Fisher Scientific), and equal amounts of total RNA were used for qRT-PCR analysis.

PPARα mRNA levels were determined by qRT-PCR using the Applied Biosystems 7500 Fast Real-Time PCR System (Applied Biosystems, Carlsbad, CA, USA) and the Applied Biosystems^®^ TaqMan^®^ RNA-to-CT™ 1-Step Kit (Thermo Fisher Scientific), following the manufacturer’s instructions. Peptidylprolyl isomerase A (PPIA) was used as a housekeeping gene. Primers for PPARα (Assay ID: Hs00947536_m1, FAM-MGB) and PPIA (Assay ID: Hs99999904_m1, VIC-MGB) were purchased from Thermo Fisher Scientific. Relative PPARα mRNA expression was calculated using the 2^−ΔΔCt^ method after normalization to PPIA and the corresponding vehicle control and expressed as percentage of vehicle control.

### 2.15. Transmission Electron Microscopy

A549 and H460 cells were plated at a density of 2.5 × 10^6^ cells per 10-cm Petri dish and cultured in serum-containing DMEM for 24 h. Subsequently, cells were treated with CBG, CBC, or vehicle in serum-free DMEM for 24 h and then collected as described for the whole-protein extraction experiments. The resulting cell pellets were fixed in a solution containing 2% glutaraldehyde and 1% paraformaldehyde in 0.1 M phosphate buffer (pH 7.3). Further processing steps correspond to an earlier study [28]. In short, the cell pellet was embedded in agarose, post-fixed with osmium tetroxide, dehydrated, and infiltrated with Epon 812 resin. After thermal curing, the resulting resin blocks were cut and 0.5 µm sections were stained with toluidine blue for histological inspection. Thin sections (50–70 nm) were mounted on copper grids and were contrasted with uranyl acetate and lead citrate for transmission electron microscopy (TEM). Images were captured using a Zeiss EM 902 TEM (Carl Zeiss, Oberkochen, Germany) equipped with a side-mounted 1 × 2k FT-CCD camera (Proscan, Scheuring, Germany) and iTEM camera control and imaging software (iTEM version number 1187, Olympus Imaging Solutions, Münster, Germany).

### 2.16. Statistics

Statistical analyses were performed using GraphPad Prism 9.3.0 or later (GraphPad Software, San Diego, CA, USA). IC_50_ values were estimated by nonlinear regression using a four-parameter logistic model. Data are presented as mean ± standard error of the mean (SEM) from independent experiments (biological replicates) performed on different days using cells from different passages.

Comparisons between two groups were performed using unpaired two-tailed Welch’s *t*-tests. For multiple group comparisons, one-way ANOVA or repeated-measures (RM) one-way ANOVA was applied, the latter corresponding to a randomized block design for matched observations [29]. Post hoc analyses were performed using Dunnett’s test for comparisons against vehicle controls, or Bonferroni correction for predefined pairwise comparisons, as appropriate. In all analyses, *p* ≤ 0.05 was considered statistically significant.

For RM (randomized block) analyses, each biological replicate was treated as a matched block, thereby accounting for inter-experimental variability and improving statistical power by reducing between-experiment variance while preserving estimation of treatment effect sizes. Statistical significance in this design depends on both effect magnitude and within-experiment consistency. Accordingly, small but highly consistent effects may reach statistical significance, whereas larger but more variable effects may not reach statistical significance, although they may still be biologically relevant.

For selected assays (cell survival, metabolic activity, caspase activity, mitochondrial superoxide levels, mitochondrial membrane potential, OCR), technical replicates were performed per biological replicate. Each technical replicate represented an independent measurement from a separate well seeded with cells derived from the same biological replicate, with treatments and assays performed independently in each well. The mean of the technical replicates was calculated for each biological replicate, and statistical analyses as well as graphical representations were performed on biological replicate means.

## 3. Results

### 3.1. CBG and CBC Decrease the Survival and Metabolic Activity of A549 and H460 Cells in a Concentration- and Time-Dependent Manner

**Figure 1:**
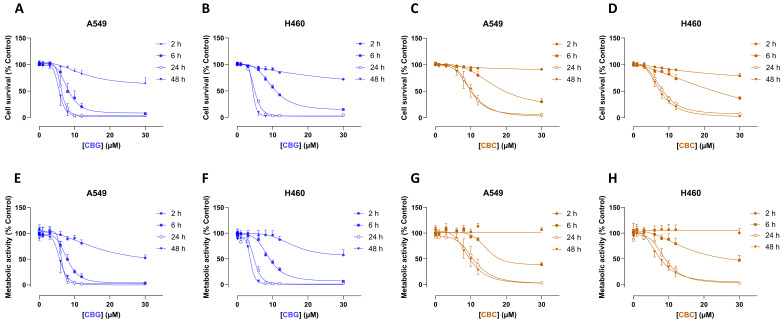
Concentration- and time-dependent effect of CBG and CBC on survival (**A**–**D**) and metabolic activity (**E**–**H**) in A549 and H460 cells. Cells were incubated with the respective cannabinoid at the indicated concentrations for the indicated times. Data were obtained using crystal violet (**A**–**D**) or MTT (**E**–**H**) assays. All percentage values shown refer to the respective time-matched vehicle control, which was set to 100%. Data represent the mean ± SEM of 4 biological replicates, each performed in technical triplicate.

To obtain initial information on the effects of CBG and CBC on the viability of the two lung cancer cell lines, the time- and concentration-dependent effects of both phytocannabinoids on the survival and metabolic activity of A549 and H460 cells were determined using crystal violet staining of viable adherent cells and the MTT test. As shown in Figure 1, both cannabinoids showed a time-dependent inhibitory effect on both parameters in A549 and H460 cells, resulting in measurable IC_50_ values within the tested concentration range of up to 30 µM after only 6 h of incubation (Supplementary Table S1). After 24 h of incubation, the IC_50_ values for CBG were around 5 to 6 µM and thus lower than those for CBC, which ranged from approximately 8 to 11 µM (Supplementary Table S1). Overall, maximum toxicity appeared to be reached after 24 h, with the inhibition curves (Figure 1) and IC_50_ values (Supplementary Table S1) for both cannabinoids being nearly identical after 24 and 48 h of incubation.

**Figure 2:**
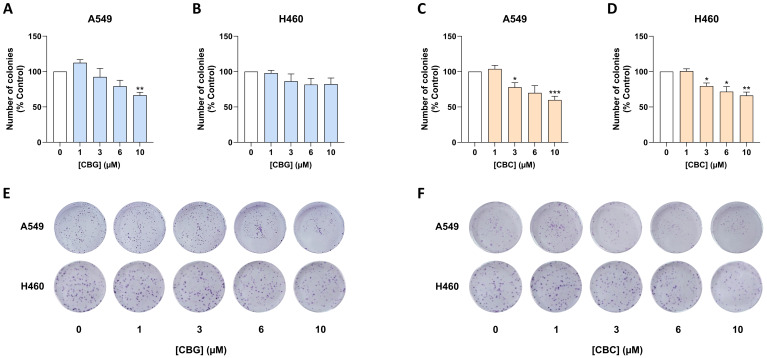
Concentration-dependent effect of CBG and CBC on colony-forming properties of A549 and H460 cells. Cells were treated with CBG (**A**,**B**,**E**), CBC (**C**,**D**,**F**) or vehicle in serum-containing DMEM for 7 days. Afterwards, cells were fixed and stained with crystal violet. Colonies were quantified as the total number of colonies per well. All percentage values shown refer to the respective vehicle control, which was set to 100%. Data represent the mean ± SEM of 6 biological replicates. For each cell line and cannabinoid, representative photographs from a single biological replicate are shown; images correspond to individual wells of a 6-well plate (well diameter, 3.5 cm; surface area, 9.5 cm^2^). * *p* ≤ 0.05, ** *p* ≤ 0.01, *** *p* ≤ 0.001 vs. corresponding vehicle control; statistical analyses were performed on raw colony counts using RM one-way ANOVA with Dunnett’s post hoc test.

In another set of experiments, the influence of CBG and CBC on the clonogenic properties of A549 and H460 cells was determined using a colony formation assay. In addition to its original purpose, which is to determine the effects of ionizing radiation therapy on in vitro cell systems [30], this test is also frequently employed to assess the long-term survival rate of tumor cells and to evaluate the effectiveness of cytostatic drugs [31]. In corresponding tests, both cannabinoids showed largely concentration-dependent suppression by up to 33% (10 µM CBG in A549 cells), 40% (10 µM CBC in A549 cells) and 34% (10 µM CBC in H460 cells), respectively (Figure 2A,C,D). In contrast, the clonogenic properties of H460 cells were less affected by CBG (Figure 2B). In A549, a slight clonogenic effect was observed at a treatment concentration of 1 µM CBG (Figure 2A), which, however, did not occur at higher concentrations.

### 3.2. CBG and CBC Reduce the Survival Rate and Metabolic Activity of A549 and H460 Cells Independently of Various Cannabinoid Membrane Receptors, but in the Case of CBG via the Transcription Factor PPARα

**Figure 3:**
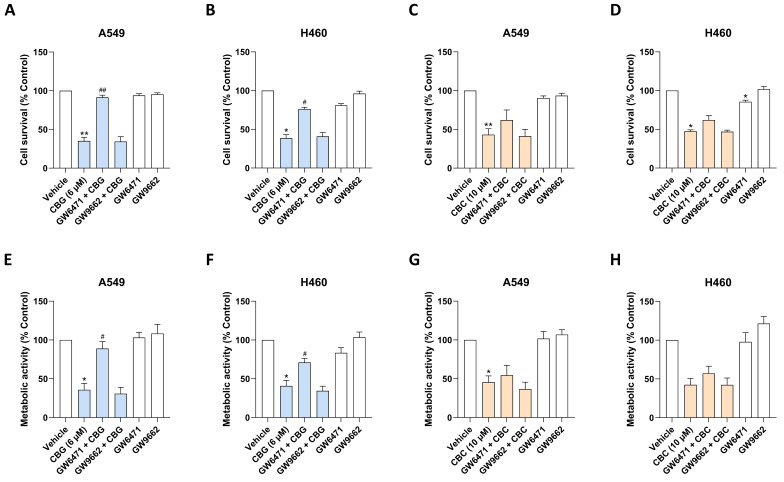
Influence of the PPARα antagonist GW6471 and the PPARγ antagonist GW9662 on the decrease in cell survival (**A**–**D**) and metabolic activity (**E**–**H**) mediated by CBG or CBC in A549 and H460 cells. Cells were pre-treated with GW6471 or GW9662 (each at 10 µM) or their vehicle for 1 h, followed by a 24 h co-incubation with CBG (6 µM) or CBC (10 µM) or their vehicle. Data represent the mean ± SEM of 4 biological replicates, each performed in technical triplicate. * *p* ≤ 0.05, ** *p* ≤ 0.01 vs. corresponding vehicle control; # *p* ≤ 0.05, ## *p* ≤ 0.01 vs. corresponding CBG-treated group; statistical analyses were performed on blank-corrected absorbance data using RM one-way ANOVA with Bonferroni’s post hoc test (pre-specified comparisons).

In order to characterize the shown regulations more precisely with regard to the initial upstream receptor targets, inhibitor experiments were carried out with antagonists against the receptors CB_1_ (AM251), CB_2_ (AM630), TRPV1 (capsazepine), and the transcription factors PPARα (GW6471) and PPARγ (GW9662). The concentrations of antagonists or inhibitors used were selected based on previous studies that documented corresponding inhibitory effects of CB_1_, CB_2_, and TRPV1 antagonists at 1 µM [32,33,34] and of GW6471 or GW9662 at 10 µM [35,36,37]. As in previous work, the antagonists did not markedly affect cell viability at the concentrations used (Figure 3, Supplementary Figure S1A–D). Cannabinoid concentrations were selected based on their ability to induce an approximately 50% reduction in viability (CBG at 6 µM, CBC at 10 µM), as shown in Figure 1. However, no reversal of the corresponding cannabinoid effects on cell survival was observed in the presence of CB_1_, CB_2_ and TRPV1 antagonists (Supplementary Figure S1A–D). Similarly, no inhibition of CBG and CBC effects and no single-application intrinsic effects of the antagonists were observed when AM251, AM630, and capsazepine were used at a concentration of 3 µM each (Supplementary Figure S2).

Based on reports describing CBG as a TRPM8 antagonist [11] and findings that TRPM8 inhibition is involved in the antiproliferative effect of CBG on colon cancer cells [38], this receptor was also investigated as a potential cannabinoid target. To this end, the effect of the TRPM8 agonists WS12 and icilin in established and efficient concentrations [39,40] was examined, which revealed no noticeable effect per se on the viability of A549 and H460 cells. However, these compounds did not affect the reduction in viability induced by either CBG or CBC (Supplementary Figure S1E–H).

On the other hand, the effects of CBG, both at the level of cell survival and metabolic activity, were significantly attenuated by the PPARα antagonist GW6471 with an almost complete reversal in A549 cells (Figure 3A,B,E,F). In the case of CBC, a slight weakening of the toxic effect was registered, albeit without significance (Figure 3C,D,G,H). In contrast, the PPARγ antagonist GW9662 was ineffective in reversing the viability reduction caused by CBG and CBC (Figure 3).

To support a PPARα-specific mechanism rather than nonspecific off-target effects, GW6471 was again tested at increasing concentrations, revealing a concentration-dependent attenuation of the CBG-induced reduction in cell survival and metabolic activity (Supplementary Figure S3).

**Figure 4:**
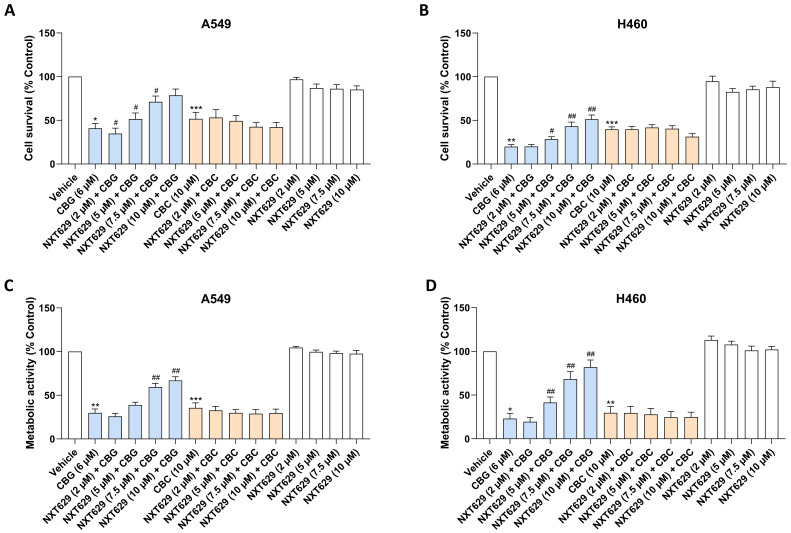
Influence of the PPARα antagonist NXT629 on the decrease in cell survival (**A**,**B**) and metabolic activity (**C**,**D**) mediated by CBG or CBC in A549 and H460 cells. Cells were pre-treated with NXT629 at the indicated concentrations or its vehicle for 1 h, followed by a 24 h co-incubation with CBG (6 µM) or CBC (10 µM) or its vehicle. Data represent the mean ± SEM of 8 biological replicates, each performed in technical triplicate. * *p* ≤ 0.05, ** *p* ≤ 0.01, *** *p* ≤ 0.001 vs. corresponding vehicle control; # *p* ≤ 0.05, ## *p* ≤ 0.01 vs. corresponding CBG- or CBC-treated group; statistical analyses were performed on blank-corrected absorbance data using RM one-way ANOVA with Bonferroni’s post hoc test (pre-specified comparisons).

Additional validation experiments were performed using the structurally distinct PPARα antagonist NXT629, which has been reported to inhibit human PPARα with an IC_50_ of approximately 77–78 nM while displaying substantially weaker activity toward other PPAR isoforms (PPARδ ~6 µM; PPARγ ~15 µM) [41,42,43], thereby indicating high selectivity for PPARα. Consistent with this pharmacological profile, NXT629 induced a concentration-dependent reversal of the CBG- but not CBC-mediated reduction in cell survival and metabolic activity (Figure 4), thus specifically confirming the involvement of PPARα signaling in mediating the effects of CBG.

**Figure 5:**
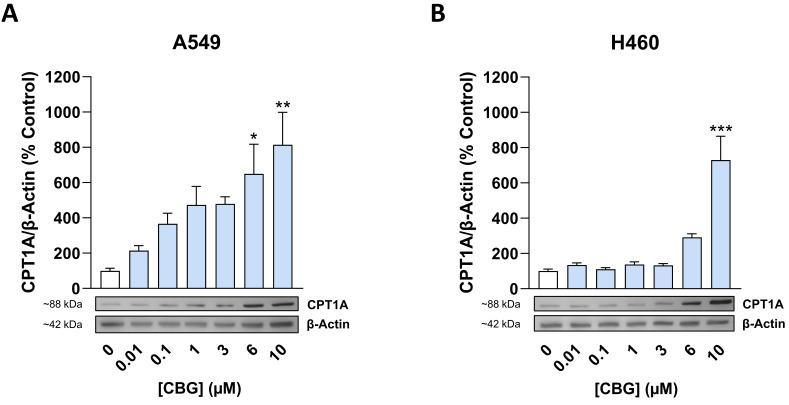
Concentration-dependent effect of CBG on CPT1A expression in A549 (**A**) and H460 cells (**B**). Cells were incubated with CBG at the indicated concentrations for 24 h. CPT1A was quantified by densitometry, normalized to β-actin, and expressed relative to the respective vehicle control (mean = 100%). The blots shown are representative. Data represent the mean ± SEM of 3 biological replicates. * *p* ≤ 0.05, ** *p* ≤ 0.01, *** *p* ≤ 0.001 vs. corresponding vehicle control; statistical analyses were performed on β-actin-normalized data expressed as percentages of the respective vehicle control using one-way ANOVA with Dunnett’s post hoc test.

As mentioned earlier, molecular docking, molecular dynamics, and luciferase assays previously identified CBG as a PPARα agonist [12]. In the present study, CBG upregulated the PPARα-dependent inducible protein carnitine palmitoyltransferase 1A (CPT1A) [44,45,46,47] in a concentration-dependent manner in both A549 and H460 cells (Figure 5), confirming PPARα activation in these cell systems. To explore a potential role of CPT1A in CBG-induced cytotoxicity, the CPT1A inhibitor etomoxir was tested. However, it did not affect the CBG-induced decrease in cell viability and metabolic activity in A549 and H460 cells, suggesting that while CPT1A serves as a marker of PPARα activation, it does not directly contribute to the cytotoxic effects of CBG in these cell lines (Supplementary Figure S4).

This study also assessed PPARα mRNA levels in A549 and H460 cells after CBG incubation. However, consistent with results obtained in HepG2 cells [12], these levels were not elevated following CBG treatment (Supplementary Figure S5), which rules out potential feedback effects. Furthermore, GW6471, alone or in combination with CBG, did not substantially affect basal PPARα mRNA expression (Supplementary Figure S5).

### 3.3. CBG and CBC Induce Apoptosis and Autophagy in A549 and H460 Cells

**Figure 6:**
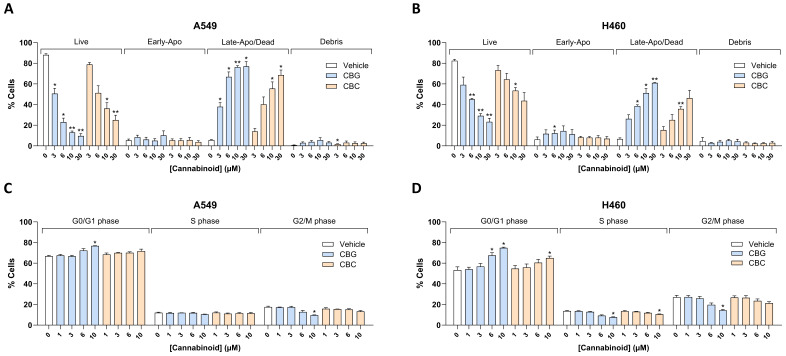
Concentration-dependent effects of CBG and CBC (24 h treatment) on early and late apoptosis (**A**,**B**) and cell cycle (**C**,**D**) in A549 and H460 cells, as determined by the Muse^®^ Annexin V & Dead Cell Kit and the Muse^®^ Cell Cycle Kit. Cells were incubated for 24 h with the respective cannabinoid at the specified concentrations or its vehicle. In panels (**A**,**B**), cells were stained using the Muse^®^ Annexin V & Dead Cell Kit to distinguish between living (Live), early apoptotic (Early-Apo), late apoptotic/dead (Late-Apo/Dead), and non-apoptotic debris. Data in panels (**C**,**D**) were obtained using the Muse^®^ Cell Cycle Kit, with propidium iodide (PI) staining to assess DNA content and distinguish between different cell cycle phases. Data represent the mean ± SEM of 3 biological replicates. * *p* ≤ 0.05, ** *p* ≤ 0.01 vs. corresponding vehicle control; statistical analyses were performed on percentages of gated cells using RM one-way ANOVA with Dunnett’s post hoc test.

To determine whether apoptosis underlies the cell death caused by CBG and CBC, the externalization of phosphatidylserine (PS) from the inner to the outer leaflet of the plasma membrane, a hallmark of apoptosis, was assessed by Annexin V staining. To distinguish between living and dead cells, this assay uses the fluorescent dye 7-AAD (7-aminoactinomycin D), which can only penetrate cells without an intact cell membrane [48]. The following positive (+) and negative (−) Annexin and 7-AAD detections are obtained for the analyzed cells: Annexin V (−) and 7-AAD (−) for non-apoptotic cells; Annexin V (+) and 7-AAD (−) for cells in early apoptosis; Annexin V (+) and 7-AAD (+) for cells in late apoptosis and dead cells; Annexin V (−) and 7-AAD (+) for non-apoptotic debris. As shown in Figure 6A,B, a 24-h incubation of both cell lines with CBG and CBC resulted in a concentration-dependent decrease in living cells, accompanied by an equally concentration-dependent increase in late apoptotic cells. After 6 h of incubation, early apoptosis increased in response to all tested concentrations of CBG and CBC, showing a pronounced concentration dependence in CBG-treated H460 cells (Supplementary Figure S6).

The percentage of total cells present at each phase of the cell cycle (G0/G1, S, and G2/M) after 24 h of cannabinoid incubation was also quantified. This showed an increase in the proportion of cells in the G0/G1 phase and a decrease in cells in the G2/M phase after incubation with both cannabinoids, with the effects being more pronounced for CBG, accompanied by significant changes in either cell line at 10 µM CBG (Figure 6C,D).

**Figure 7:**
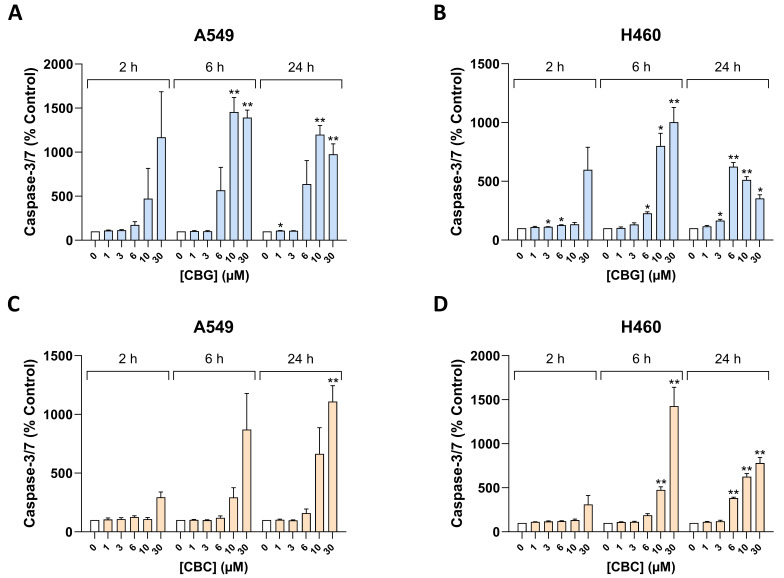
Concentration- and time-dependent effect of CBG (**A**,**B**) and CBC (**C**,**D**) on caspase-3/7 activity in A549 and H460 cells. Cells were incubated with the respective cannabinoid at the indicated concentrations for the indicated times. Data were obtained using caspase-3/7 activity assays. All percentage values shown refer to the respective time-matched vehicle control, which was set to 100%. Data represent the mean ± SEM of 3 biological replicates, each performed in technical triplicate. * *p* ≤ 0.05, ** *p* ≤ 0.01 vs. corresponding vehicle control; statistical analyses were performed on log-transformed, blank-corrected luminescence data using RM one-way ANOVA with Dunnett’s post hoc test.

To obtain additional evidence of apoptotic cell death, the effect of CBG and CBC on the activity of executioner caspases-3 and -7 was investigated (Figure 7). Concentration-dependent induction was detected for both cannabinoids at three different time points, with 6 µM CBG, which lies within the IC_50_ cytotoxicity range (Figure 1, Supplementary Table S1), already resulting in 5.7-fold (A549) and 2.3-fold (H460) inductions after 6 h of incubation (Figure 7A,B). Even at approximately half-maximal cytotoxic concentrations of 10 µM CBC, increases in activity of 2.9-fold (A549) and 4.8-fold (H460) were already observed at this time point (Figure 7C,D).

**Figure 8:**
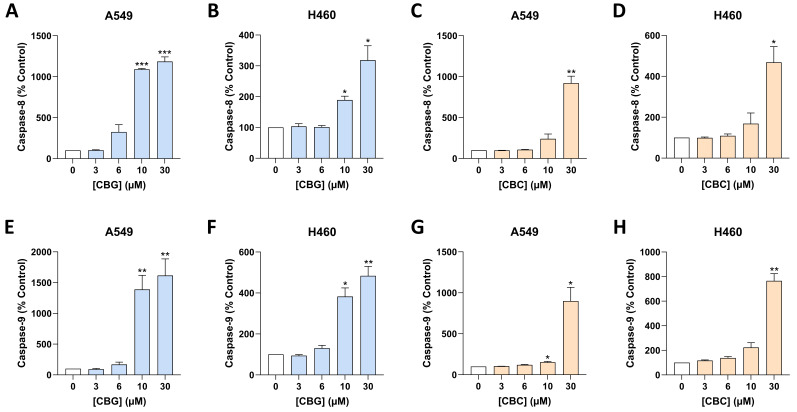
Concentration-dependent effect of CBG and CBC on activation of caspase-8 (**A**–**D**) and caspase-9 (**E**–**H**) in A549 and H460 cells. Cells were incubated with CBG or CBC at the indicated concentrations for 6 h. Data were obtained using caspase activity assays. All percentage values shown refer to the respective time-matched vehicle control, which was set to 100%. Data represent the mean ± SEM of 3 biological replicates, each performed in technical triplicate. * *p* ≤ 0.05, ** *p* ≤ 0.01, *** *p* ≤ 0.001 vs. corresponding vehicle control; statistical analyses were performed on log-transformed, blank-corrected luminescence data using RM one-way ANOVA with Dunnett’s post hoc test.

**Figure 9:**
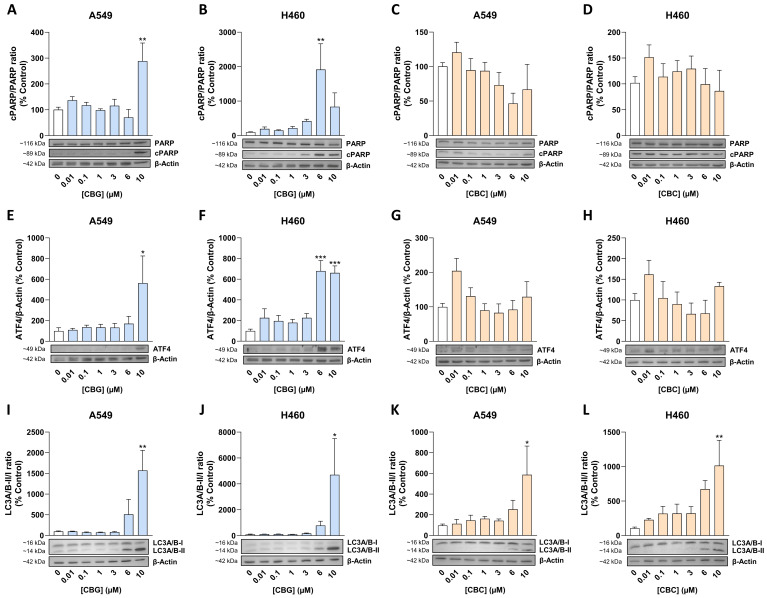
Concentration-dependent effect of CBG and CBC on PARP cleavage, yielding cleaved PARP (cPARP; **A**–**D**), ATF4 expression (**E**–**H**), and conversion of LC3-I to LC3-II (**I**–**L**) in A549 and H460 cells. Cells were incubated with CBG or CBC at the indicated concentrations for 24 h. Bar graphs show densitometric analysis of the blots. cPARP, PARP, LC3A/B-I, LC3A/B-II, and ATF4 were normalized to β-actin. ATF4 levels and cPARP/PARP and LC3A/B-II/LC3A/B-I ratios are expressed relative to the respective vehicle control (mean = 100%). Representative blots are shown. Data represent the mean ± SEM of 3 biological replicates. * *p* ≤ 0.05, ** *p* ≤ 0.01, *** *p* ≤ 0.001 vs. corresponding vehicle control; statistical analyses were performed on normalized data expressed as percentages of the respective vehicle control using one-way ANOVA with Dunnett’s post hoc test.

In further experiments, early activation of the two initiator caspases-8 (Figure 8A–D) and -9 (E–H) by CBG and CBC was also demonstrated after 6 h of incubation of both cell lines examined. With reference to the previously shown induction of caspase-3/7 by 6 µM CBG and 10 µM CBC after 6 h (Figure 7), an increase in the activities of caspase-8 and caspase-9 was also observed at this time point upon treatment with the corresponding cannabinoid concentrations, as expected, with only one exception (Figure 8B). Overall, CBG and CBC showed a clear concentration-dependent induction of both caspase-8 and caspase-9 in both cell lines.

Investigation of the cleavage of poly(ADP-ribose) polymerase (PARP), a downstream target of effector caspases, revealed significant upregulation at 10 µM CBG in A549 cells and at 6 µM CBG in H460 cells (Figure 9A,B), while no significant induction was observed in the presence of CBC (Figure 9C,D). A similar pattern was observed in the analysis of ATF4 (activating transcription factor 4), a stress-responsive transcription factor mediating autophagy and endoplasmic reticulum (ER) stress-induced apoptosis, with significant upregulation by CBG in A549 cells at 10 µM and in H460 cells at 6 and 10 µM (Figure 9E,F) and comparatively lower or non-significant induction by CBC at equimolar concentrations (Figure 9G,H).

A significant increase in the autophagy marker microtubule-associated protein 1 light chain 3-II (LC3-II) was finally detected for each of the two phytocannabinoids at a concentration of 10 µM in both lung cancer cell lines examined (Figure 9I–L).

### 3.4. CBG Induces Apoptosis of A549 and H460 Cells in a PPARα-Dependent Manner

**Figure 10:**
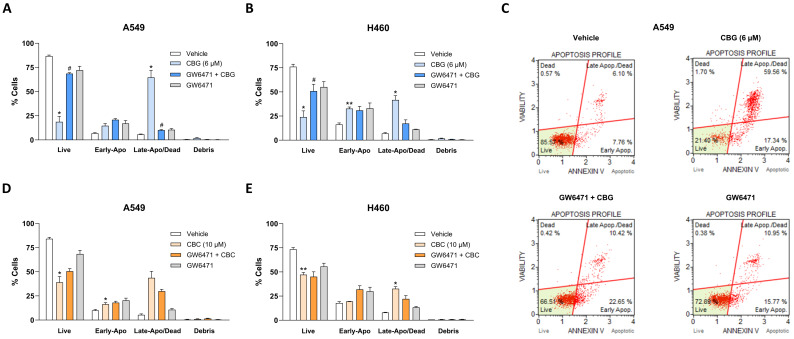
Influence of the PPARα antagonist GW6471 on total apoptotic cell fraction following treatment of A549 (**A**,**C**,**D**) and H460 cells (**B**,**E**) with CBG or CBC. Cells were pre-treated with GW6471 (10 µM) or its vehicle for 1 h, followed by a 24 h co-incubation with CBG (6 µM) or CBC (10 µM) or its vehicle. The cells were then stained using the Muse^®^ Annexin V & Dead Cell Kit to distinguish between living (Live), early apoptotic (Early-Apo), late apoptotic/dead (Late-Apo/Dead), and non-apoptotic debris. Data represent the mean ± SEM of 3 biological replicates. * *p* ≤ 0.05, ** *p* ≤ 0.01 vs. corresponding vehicle control; # *p* ≤ 0.05 vs. corresponding CBG-treated group; statistical analyses were performed on percentages of gated cells using RM one-way ANOVA with Bonferroni’s post hoc test (pre-specified comparisons).

To investigate the involvement of PPARα in the context of apoptosis, selected apoptosis markers elevated by CBG and CBC were examined by pre-incubation of cells with GW6471. Under these circumstances, the increase in apoptotic cells and decrease in viable cells induced by CBG was substantially antagonized by GW6471, with GW6471 alone leading to an increase in apoptotic A549 and H460 cells (Figure 10A,B). Exemplary plots with quadrant markings for quantitative determination of the individual cell populations are shown in Figure 10C for A549 cells.

As observed above, GW6471 showed no significant antagonistic effect on the decrease in living cells caused by CBC (Figure 10D,E). Notably, GW6471 partially inhibited the CBC-induced increase in late apoptotic/dead cells (Figure 10D,E), although this effect was quantitatively weaker than its protective effect in CBG-treated cells (Figure 10A,B).

**Figure 11:**
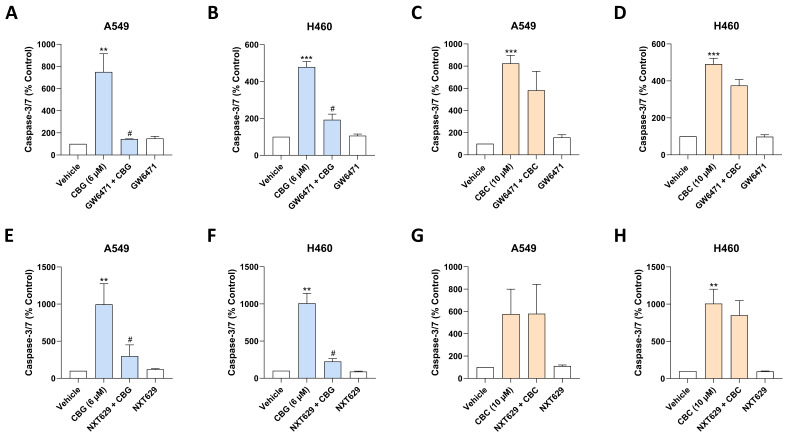
Influence of the PPARα antagonists GW6471 (**A**–**D**) and NXT629 (**E**–**H**) on caspase-3/7 activity in A549 and H460 cells following treatment with CBG or CBC. Cells were pre-treated with GW6471 (10 µM) or NXT629 (10 µM) or their vehicle for 1 h, followed by a 24 h co-incubation with CBG (6 µM) or CBC (10 µM) or their vehicle. Afterwards, the cells were analyzed using caspase-3/7 activity assays. Percentage values refer to the respective time-matched vehicle control, which was set to 100%. Data represent the mean ± SEM of 4 biological replicates, each performed in technical triplicate. ** *p* ≤ 0.01, *** *p* ≤ 0.001 vs. corresponding vehicle control; # *p* ≤ 0.05 vs. corresponding CBG-treated group; statistical analyses were performed on log-transformed, blank-corrected luminescence data using RM one-way ANOVA with Bonferroni’s post hoc test (pre-specified comparisons).

Significant inhibitory effects of the PPARα antagonists GW6471 and NXT629 were detected for CBG-induced caspase-3/7 activity (Figure 11A,B,E,F). Consistent with the viability data (Figure 3C,D,G,H) and apoptosis data (Figure 10D,E), GW6471 slightly attenuated CBC-induced caspase-3/7 activity (Figure 11C,D), without reaching statistical significance. In the case of NXT629, no notable effect on CBC-induced caspase-3/7 activation was observed (Figure 11G,H).

In H460 cells, GW6471 was additionally found to significantly reverse the CBG-upregulated LC-3-I to LC-3-II conversion, an established autophagy marker (Supplementary Figure S7B). However, this GW6471 effect was only weakly detectable in CBG-treated A549 cells, although it should be noted as a limitation that GW6471 per se caused a doubling of basal LC-3-II formation in this cell line (Supplementary Figure S7A).

### 3.5. CBG and CBC Increase Mitochondrial Superoxide, Decrease Mitochondrial Membrane Potential and Increase Cytosolic Cytochrome c Levels in A549 and H460 Cells

**Figure 12:**
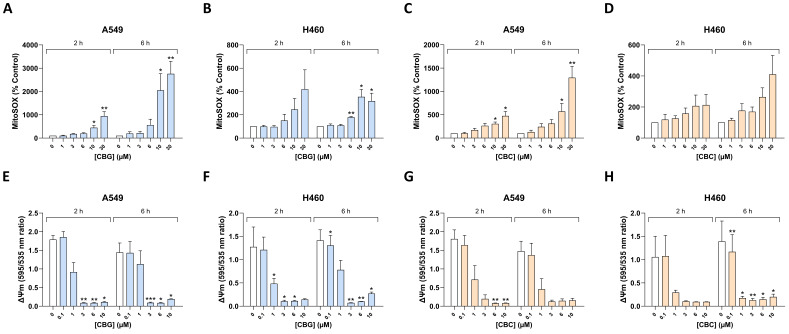
Concentration- and time-dependent effect of CBG and CBC on mitochondrial superoxide (**A**–**D**) and changes in mitochondrial membrane potential (**E**–**H**) of A549 and H460 cells. Cells were incubated with CBG or CBC at the indicated concentrations for 24 h. Data were obtained using fluorescence measurements, as described in Section 2.8 and Section 2.9. Data represent the mean ± SEM of 4 (**A**–**D**) or 3 (**E**–**H**) biological replicates, each performed in technical triplicate. Percentage values in (**A**–**D**) refer to the respective vehicle control, which was set to 100%. * *p* ≤ 0.05, ** *p* ≤ 0.01, *** *p* ≤ 0.001 vs. corresponding vehicle control; statistical analyses were performed on log-transformed fluorescence ratios derived from blank-corrected fluorescence values using RM one-way ANOVA with Dunnett’s post hoc test.

To investigate mitochondrial involvement in cannabinoid-induced apoptotic cell death, subsequent investigations focused on mitochondrial alterations elicited by CBG and CBC. To this end, MitoSOX^TM^ Red, a cell-permeable fluorogenic dye for selective detection of superoxide in mitochondria of live cells [49,50], was used in an initial experimental approach to detect mitochondrial superoxide. As shown in Figure 12A–D, increased mitochondrial superoxide levels were triggered by CBG and CBC in a concentration- and time-dependent manner in both A549 and H460 cells. In Figure 12F,H (both at 6 h), the statistical significance observed at 0.1 µM CBG and 0.1 µM CBC likely reflects the low inter-experimental variability relative to the small effect size within the randomized block ANOVA design rather than a biologically meaningful effect (see Section 2.16).

To measure changes in mitochondrial membrane potential, JC-10, a lipophilic cationic dye that can selectively penetrate mitochondria, was used (Figure 12E–H). Here, a concentration-dependent reduction in membrane potential was observed for each cannabinoid in both cell lines as early as 2 h after addition, with corresponding effects already evident at concentrations that were non-toxic (CBG at 1 and 3 µM, CBC at 1 µM) or minimally toxic (CBC at 3 µM) in the MTT or crystal violet test performed after 24 h of incubation (comp. with Figure 1). Remarkably, CBC showed slightly higher efficiency than CBG in this assay.

**Figure 13:**
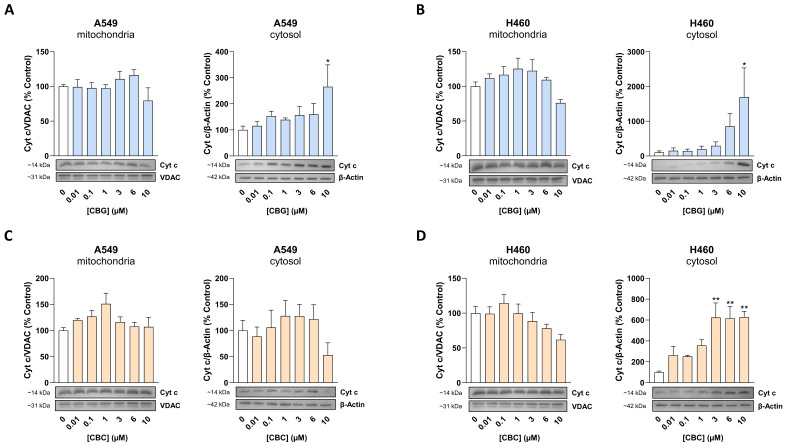
Concentration-dependent effect of CBG (**A**,**B**) and CBC (**C**,**D**) on the release of mitochondrial cytochrome c (Cyt c) into the cytosol of A549 and H460 cells. Cells were incubated with CBG or CBC at the indicated concentrations for 24 h. Bar chart values were derived from densitometric analyses of the blots. Cyt c levels were normalized on VDAC (mitochondrial fraction) and β-actin (cytosolic fraction). All percentages refer to the respective vehicle control (mean = 100%). The blots shown are representative and originate from partially different biological replicates when comparing cytosolic and mitochondrial fractions in a panel. In (**D**) the same VDAC blot is shown as in Supplementary Figure S10D, as the same membranes were stripped and reprobed for different target proteins. Data represent the mean ± SEM of 3 biological replicates. * *p* ≤ 0.05, ** *p* ≤ 0.01 vs. corresponding vehicle control; statistical analyses were performed on VDAC- and β-actin-normalized data expressed as percentages of the respective vehicle control using one-way ANOVA with Dunnett’s post hoc test.

Finally, after an incubation period of 24 h, concentration-dependent increases in mitochondrial cytochrome c were detected in the cytosolic fractions of cells treated with CBG (Figure 13A,B) or CBC (Figure 13D). In the case of no apparent effect of CBC in A549 cells (Figure 13C), corresponding analyses were repeated after 2 h of incubation, whereupon significantly increased concentrations of cytosolic cytochrome c were also detected here (Supplementary Figure S8).

### 3.6. CBG and CBC Induce Mitochondrial Structural Changes in A549 and H460 Cells, as Shown by Transmission Electron Microscopy

**Figure 14:**
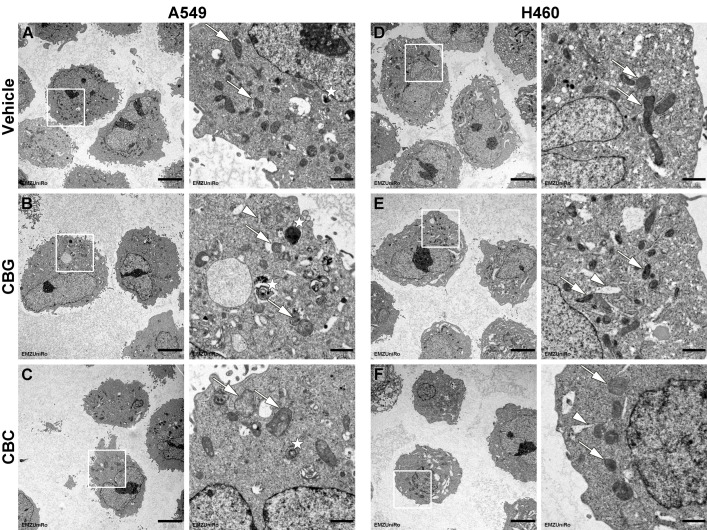
Effects of CBG and CBC treatments on the morphology and organelle structure of A549 and H460 cells. Representative transmission electron microscopy images of A549 cells (**A**–**C**) and H460 cells (**D**–**F**) treated with vehicle (**A**,**D**), 6 µM CBG (**B**,**E**), or 10 µM CBC (**C**,**F**) for 24 h. Overview images with marked areas of interest are shown in the left column, and the corresponding cellular details of these areas are shown in the images in the right column for each treatment group. Some exemplary, concise autophagosomes were marked with asterisks. In the vehicle-treated cells (**A**,**D**), numerous regularly shaped, intact mitochondria (white arrows) with adjacent rough endoplasmic reticulum (rER) membranes can be seen. In contrast, structurally altered mitochondria were observed in CBG-treated cells (**B**,**E**), including damaged mitochondrial cristae, the formation of autophagosomes (asterisks), and a mild displacement of the mitochondria-associated membranes (MAM) (white arrowheads). Similarly, after treatment with CBC (**C**,**F**), mitochondrial damage was observed, manifested in altered cristae and swollen mitochondria, but with less prominent autophagosomes and MAM displacement. The scale bars are 5 µm in the overview images and 1 µm in the detailed images.

To investigate changes in mitochondrial morphology, electron microscopic images of A549 and H460 cells were taken. In cells incubated with vehicle, most mitochondria showed a regular intact structure with numerous cristae (white arrows) and were in close contact with the rough endoplasmic reticulum (rER) (Figure 14A,D). In contrast, A549 cells treated with CBG exhibited mitochondria with a severely altered structure, indicating considerable mitochondrial damage, such as alterations in cristae organization. Condensed mitochondria (white arrows) and the formation of autophagosomes (asterisks) were also observed (Figure 14B). In the H460 cells treated with CBG, mitochondria showed less damage at the cristae (Figure 14E, white arrows). However, in both cell lines the associated rER was affected by these structural alterations, and the mitochondria-associated membranes (MAM) were widened or displaced (Figure 14B,E, white arrowhead). With CBC treatment, damage to the mitochondrial cristae and swelling of the mitochondria were prominent in both A549 and H460 cells (Figure 14C,F, white arrows). In addition, autophagosomes (asterisk) and enlarged MAM (arrowhead) were visible in some areas (Figure 14F).

### 3.7. CBG and CBC Suppress Mitochondrial Respiration in A549 and H460 Cells

**Figure 15:**
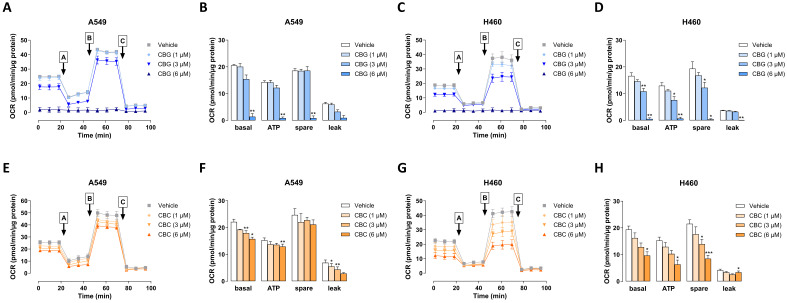
Concentration-dependent effect of CBG (**A**–**D**) and CBC (**E**–**H**) on oxygen consumption rates (OCR) in A549 and H460 cells. Cells were incubated with CBG or CBC at the indicated concentrations for 24 h. A mitochondrial stress test was then carried out and OCR values were determined using the Seahorse XFe24 Analyzer. Therefore, oligomycin (port A), FCCP (port B) and antimycin A/rotenone (port C) were loaded into the respective ports of the sensor cartridges and released into the wells at the specified times. From this assay, the time courses of OCR (**A**,**C**,**E**,**G**) in both cell lines treated with CBG or CBC are shown, and calculations (**B**,**D**,**F**,**H**) of basal respiration (basal), ATP-linked respiration (ATP), spare respiratory capacity (spare) and proton leak. Data represent the mean ± SEM of 3 biological replicates, performed in technical duplicates to quadruplicates. * *p* ≤ 0.05, ** *p* ≤ 0.01, *** *p* ≤ 0.001 vs. corresponding vehicle control; statistical analyses were performed on protein-normalized OCR values using RM one-way ANOVA with Dunnett’s post hoc test.

In the next set of experiments, the oxygen consumption rate (OCR) was determined as an indicator of mitochondrial respiration. To this end, a mitochondrial stress test was performed, which quantifies basal respiration, ATP-linked respiration, spare respiratory capacity and proton leak. This revealed a CBG- and CBC-triggered concentration-dependent decrease in all parameters mentioned in both cell lines, albeit to varying degrees (Figure 15B,D,F,H). Thus, at 6 µM, CBG led to a substantially stronger inhibition of all calculated OCR values (Figure 15B,D) than equimolar concentrations of CBC (Figure 15F,H). Inhibition of basal OCR was already detectable at non-toxic concentrations of 3 µM CBG (comp. with Figure 1). In Figure 15F,H (rightmost four-bar sets), statistical significance was not observed at 6 µM CBC in Figure 15F or at 3 µM CBC in Figure 15H, although these groups showed the strongest numerical effects, indicating that significance in the randomized block ANOVA depended strongly on the consistency of within-experiment responses in addition to the magnitude of the mean effect (see Section 2.16).

To verify whether the downregulation of various OCR parameters observed in the presence of 6 µM CBG in both cell lines is caused by the activation of PPARα, mitochondrial stress tests were also performed using the PPARα antagonist GW6471, which was administered to the cells in combination with CBG or alone. Thereby, pronounced inhibitory effects of the antagonist per se on basal respiration, ATP-linked respiration, spare respiratory capacity, and proton leak were observed, although these were quantitatively lower than the corresponding effects of CBG alone (Supplementary Figure S9B,D). Despite this intrinsic effect, GW6471 in combination with CBG in A549 cells led to a measurable attenuation of the CBG effect on the four OCR parameters mentioned above (Supplementary Figure S9B). On the other hand, the attenuation of the CBG action by GW6471 was less pronounced in H460 cells and was evident here at the level of basal respiration and proton leak (Supplementary Figure S9D).

### 3.8. CBG Reduces the Mitochondrial Concentration of NDUFB8, a Subunit of Respiratory Chain Complex I, in A549 and H460 Cells

**Figure 16:**
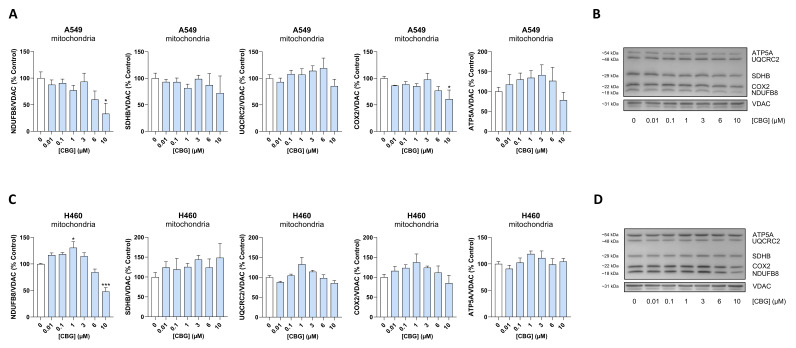
Concentration-dependent effect of CBG on the concentrations of subunits of mitochondrial respiratory chain complexes in A549 (**A**,**B**) and H460 cells (**C**,**D**). Cells were treated with the indicated concentrations of CBG for 24 h. Thereafter, the corresponding proteins in the mitochondrial fractions were determined using Western blot analysis. Bar chart values were derived from densitometric analyses of the blots. Mitochondrial proteins were normalized to VDAC. All percentages refer to the respective vehicle control (mean = 100%). The blots shown are representative. Data represent the mean ± SEM of 3 biological replicates. * *p* ≤ 0.05, *** *p* ≤ 0.001 vs. corresponding vehicle control; statistical analyses were performed on VDAC-normalized data expressed as percentages of the respective vehicle control using one-way ANOVA with Dunnett’s post hoc test.

In the next step, the influence of CBG and CBC on the concentrations of specific subunits of the respiratory chain complexes in protein extracts from isolated mitochondria was addressed using an OxPhos antibody set. Specifically, NADH:ubiquinone oxidoreductase subunit B8 (NDUFB8, subunit of complex I), succinate dehydrogenase complex iron-sulfur subunit B (SDHB, subunit of complex II), ubiquinol-cytochrome c reductase core protein 2 (UQCRC2, subunit of complex III), cytochrome c oxidase subunit 2 (COX2, subunit of complex IV) and mitochondrial ATP synthase F1 subunit alpha (ATP5A) were analyzed. Noteworthy here is a significant reduction in NDUFB8 of more than 50% in the mitochondrial fractions of A549 (Figure 16A,B) and H460 cells (Figure 16C,D) treated with 10 µM CBG for 24 h. In contrast, quantitatively comparable reductions in respiratory chain proteins were not observed in CBC-treated A549 or H460 cells (Supplementary Figure S10). Nevertheless, CBC led to a moderate decrease in certain proteins, which was statistically significant for UQCRC2 in H460 cells (Supplementary Figure S10).

## 4. Discussion

The present study demonstrates the viability-reducing effects of the non-psychoactive phytocannabinoids CBG and CBC on non-small cell lung cancer cells, highlighting the significant involvement of mitochondrial-induced apoptosis and mitochondrial dysfunction in this process. There are several lines of evidence to support this conclusion. First, in various assays, both cannabinoids caused a time- and concentration-dependent reduction in the viability of two established cell lines (A549, H460) of this tumor entity, as reflected by decreased numbers of adherent cells (crystal violet) and reduced mitochondrial metabolic activity (MTT), indicating impairment of both cell number and cellular metabolic function. Second, this process was accompanied by activation of the initiator caspases-8 and -9, increased activity of the effector caspases-3 and -7, and increased Annexin V positivity on the cell surface, all of which are established indicators of apoptosis, as well as increased formation of the autophagy marker LC3-II. Third, in line with the activation of caspase-9, an increased release of mitochondrial cytochrome c into the cytosol and a reduction in mitochondrial membrane potential were observed. Fourth, in appropriately treated cells, the mitochondrial dysfunction caused by both cannabinoids was further confirmed by increased mitochondrial superoxide formation, structural changes in the mitochondria visible under electron microscopy, a decrease in OCR, and, in the case of CBG, a reduction in the respiratory chain complex I subunit NDUFB8. Concerning electron microscopy analyses, CBG induced widening and displacement of oxidative-stress–sensitive MAMs [51], which likewise aligns with the CBG-triggered oxidative stress, autophagy and mitochondrial apoptosis.

The effect of CBG and CBC against cancer cells is well known and was tested on a number of tumor cell lines by Ligresti et al. as early as 2006 [15], with CBG showing greater efficacy than CBC, in line with our data. Since then, both cannabinoids have also been demonstrated to induce apoptosis, with CBG proving effective, for example, in cholangiocarcinoma [52], colorectal carcinoma [53], glioblastoma [54], mesothelioma [55] and prostate carcinoma cells [56]. In the case of CBC, pro-apoptotic effects have been described, for example, in bladder cancer [57], colon carcinoma [58] and pancreatic carcinoma cells [59].

To our knowledge, this is the first study to demonstrate that the pro-apoptotic effect of CBG on cancer cells is associated with activation of the transcription factor PPARα. As already mentioned, increased PPARα activity in cancer cells has been linked to antiproliferative [17], cytotoxic [18,19] and mitochondrial dysfunction-promoting [20,21] effects. In the case of cannabinoids, PPARα dependence of cytotoxic events has so far been demonstrated for THC, CBD and the combination of THC and CBD in melanoma cells [60].

In the present study, the PPARα inhibitors GW6471 and NXT629 significantly reversed the CBG-mediated reduction in cell viability, reflected by changes in cell survival rate and metabolic activity. Moreover, GW6471 suppressed the CBG-associated increase in apoptosis markers, including phosphatidylserine externalization, as measured by Annexin V binding. Consistently, CBG-induced caspase-3/7 activity was reduced by both GW6471 and NXT629. Western blot analyses clearly showed a concentration-dependent induction of the PPARα-dependent protein CPT1A [44,45,46,47] by CBG in both cell lines, which can be regarded as evidence of PPARα activation. Finally, no comparable PPARα-dependent cell death and apoptosis effects were observed in the CBC tests conducted in parallel, although minor changes were detected. It is noteworthy that CBG, together with cannabidiolic acid (CBDA) and cannabigerolic acid (CBGA), has previously been identified as a dual PPARα/γ agonist [12]. However, the PPARγ antagonist GW9662 did not inhibit the viability-reducing and pro-apoptotic action of CBG in the present study, which rules out a significant contribution of PPARγ to the CBG effects investigated here.

With regard to other antagonists tested on the CB_1_ and CB_2_ receptors or on TRPV1, no evidence was found for the involvement of these membrane receptors in CBG- or CBC-induced cell death. In an earlier study conducted on colorectal cancer cells, the antiproliferative effect of CBG was mimicked by TRPM8 antagonists and reduced in TRPM8-silenced cells [38]. However, in our hands, the two TRPM8 agonists WS12 and icilin showed no effect on CBG- and CBC-induced lung cancer cell death, which argues against the involvement of TRPM8 in these processes.

Special mention should also be made of individual parameters recorded in the present study. Thus, at the time of Annexin V staining to detect apoptosis, the cell cycle was also determined, which showed percentage increases in the G0/G1 phase and decreases in the G2/M phase for CBG and CBC. In colorectal cells, similar results were recently reported for CBG at 30 µM following 24 h incubation, with increased phosphatidylserine externalization, as indicated by Annexin V binding, accompanied by G1 arrest and a reduced percentage of cells in G2/M phase [53]. G1 arrest has also been described for other cannabinoids in tumor cells, including prostate cancer [61] and gastric cancer cells [62].

The early induction of two initiator caspases—caspase-8, a mediator of the extrinsic pathway, and caspase-9, a mediator of the intrinsic pathway—by CBG and CBC in both cell lines was surprising, yet consistent with the effects of other cannabinoids in various cancer cell lines [63,64,65,66]. It is also known that both initiator caspases not only activate effector caspases-3 and -7 in a synergistic manner, but that the cleavage of BID, a member of the Bcl-2 family, mediated by caspase-8 is also involved in the release of cytochrome c as part of the intrinsic signaling pathway [67,68].

Interestingly, a significant cleavage of the caspase substrate PARP was detected for CBG, but not for CBC. In the latter case, this could be due to missing the optimal time for detection or to a general absence of this reaction. Whatever the cause, cases of early and late apoptosis have been described in the past, characterized by increased Annexin V staining, mitochondrial bifurcations, and a reduction in mitochondrial membrane potential, but without PARP cleavage [69].

Finally, we were able to show that both CBG and CBC led to the activation of autophagy in A549 and H460 cells, as measured by an upregulation of LC3-II, a standard marker for autophagosomes. Indeed, autophagosomes associated with mitochondria were abundant in the electron microscopic analysis of cannabinoid-treated A549 cells. In the case of CBG, the LC3-II induction in H460 cells also appears to be linked to the activation of PPARα, as the LC3-II increase caused by CBG was significantly reduced by the PPARα inhibitor GW6471. This seems to be a cell type-specific reaction that was only very weakly observed in A549 cells. Autophagy is attributed to cell protection, but it can also play a cytotoxic role in the sense of non-canonical autophagy-mediated apoptosis, as has been shown, for example, for the toxic effect of THC on melanoma cells [70].

Treatment with higher concentrations of CBG, alongside the observed changes in LC3-II, also induced a notable upregulation of ATF4 in the studied cells. ATF4 is a stress-induced transcription factor with a highly context-dependent role in regulating cell fate. Depending on the cellular context, it may support cancer cell survival, trigger apoptosis, or induce autophagy (for review, see [71]). In the present study, potential downstream pathways mediating the effects of ATF4 induction were not directly explored.

A functional analysis of mitochondrial respiration ultimately revealed that both CBG and CBC led to concentration-dependent inhibition of basal respiration, ATP-linked respiration, spare respiratory capacity, and proton leak in both A549 and H460 cells. CBG elicited a greater reduction in all calculated OCR values than CBC and triggered significant inhibition of basal OCR even at non-toxic concentrations. Furthermore, at the highest concentration tested, CBG caused a more than 50% reduction in the protein concentrations of NDUFB8, a subunit of respiratory chain complex I. An earlier investigation by our group also described a reduction in complex I subunit in glioblastoma cells through the combination of THC and CBD [72]. Literature data on the influence of CBG and CBC on oxidative phosphorylation using an extracellular flow analyzer are scarce. When testing a concentration of 3 µM CBG in hormone-refractory prostate cancer cells [73], a tendency toward increased OCR was observed, while CBD at 3 µM significantly inhibited the maximum capacity of oxidative phosphorylation under the same conditions.

Previous studies on the effect of PPARα activation on mitochondrial respiration have yielded varying and sometimes contradictory results. On the one hand, PPARα stimulates genes for fatty acid uptake and β-oxidation, thereby also providing more substrates for mitochondrial respiration, accompanied by an increase in basal OCR, ATP-bound OCR, and reserve respiratory capacity when fatty acids are available [74]. On the other hand, PPARα agonists can also reduce OCR when glycolysis is simultaneously suppressed or apoptosis is induced [20,75]. In one of these studies, the PPARα activator fenofibrate was shown to act primarily by inhibiting complex I of the respiratory chain [76], which is consistent with the CBG-mediated downregulation of NDUFB8 described above. In the present study, PPARα inhibition with GW6471 led to a partial, non-significant reversal of the inhibitory effect of CBG on basal respiration, ATP-linked respiration, spare respiratory capacity, and proton leak in A549 cells, suggesting at least a certain role for PPARα in this process. These reversal effects were recorded despite the pronounced inhibitory effects of GW6471 on the aforementioned respiration parameters when applied to the cells alone. Remarkably, both effects are also found in the literature. Thus, the basal respiration, ATP-linked respiration, and maximum respiration reduced by the PPARα agonist fenofibrate in gastric cancer cells were reversed by PPARα siRNA [20]. In human glioblastoma cells, however, PPARα siRNA did not prevent the OCR reduction caused by fenofibrate, and GW6471 caused only minimal reversal [77]. With GW6471 alone, OCR reductions were observed at 25 µM in renal carcinoma cells [78] and at 5 µM in ovarian cancer stem cells [79], while a slight increase in OCR was found at 1 µM in human glioblastoma cells [77]. In summary, the effect of both PPARα activation and inhibition on mitochondrial respiration is context-dependent and requires further in-depth analysis, in this case particularly with regard to the effect of CBG.

This study has several limitations that should be considered. First, unlike CBG, no initial receptor for CBC could be identified that triggers the pro-apoptotic signaling cascade under the conditions used. While CBC-induced cytotoxicity has been linked to CB_2_ and TRPV1 in pancreatic cancer cells [59], these receptors did not appear to mediate the effects observed in our model, pointing to potential cell type–specific differences. Second, CBG-induced PPARα activation was assessed indirectly by measuring CPT1A induction and by pharmacological inhibition using different antagonists, including the highly selective antagonist NXT629 [41,42,43]. Although widely applied, this approach does not fully substitute for direct functional assays. Prior studies have demonstrated binding of CBG to the PPARα ligand-binding domain through molecular modeling and confirmed functional receptor activation in luciferase reporter assays [12]. Extending such approaches to lung cancer models would further strengthen mechanistic insight. Third, complementary genetic approaches, such as siRNA-mediated knockdown, could further corroborate the PPARα-dependent effects of CBG.

## 5. Conclusions

The non-psychoactive phytocannabinoids CBG and CBC, which remain comparatively underexplored, were shown to induce pronounced pro-apoptotic effects and mitochondrial dysfunction in the human lung cancer cell lines A549 and H460, with CBG acting via the transcription factor PPARα to promote apoptotic cell death. Notably, CBG exerted stronger effects than CBC with regard to cell viability reduction and inhibition of mitochondrial respiration, suggesting greater translational relevance for therapeutic development. The involvement of PPARα in CBG-mediated cytotoxicity warrants further investigation in preclinical models and across additional tumor entities.

## Data Availability

The original contributions presented in this study are included in the article/Supplementary Materials. Further inquiries can be directed to the corresponding author.

## Supplementary Materials

The following supporting information can be downloaded at: https://www.mdpi.com/article/10.3390/antiox15060754/s1. Table S1. Concentration-dependent effects of CBG and CBC on survival and metabolic activity in A549 and H460 cells incubated with CBG or CBC for different periods of time. Figure S1. Influence of antagonists of CB_1_ (AM251), CB_2_ (AM630), and TRPV1 (capsazepine, CPZ) (A–D), as well as agonists of TRPM8 (WS12, icilin) (E–H), on the decrease in cell survival mediated by CBG or CBC in A549 and H460 cells. Figure S2. Influence of antagonists of CB_1_ (AM251), CB_2_ (AM630), and TRPV1 (capsazepine, CPZ), each at 3 µM, on the decrease in cell survival mediated by CBG (A,B) or CBC (C,D) in A549 and H460 cells. Figure S3. Influence of the PPARα antagonist GW6471 on the decrease in cell survival (A,B) and metabolic activity (C,D) mediated by CBG in A549 and H460 cells. Figure S4. Influence of the CPT1A antagonist etomoxir on the decrease in cell survival (A,B) and metabolic activity (C,D) mediated by CBG in A549 and H460 cells. Figure S5. Effect of CBG and the PPARα antagonist GW6471, tested alone or in combination, on PPARα mRNA expression in A549 (A) and H460 cells (B). Figure S6. Concentration-dependent effects of CBG and CBC (6 h treatment) on early and late apoptosis in A549 (A) and H460 cells (B), as determined by the Muse^®^ Annexin V & Dead Cell Kit. Figure S7. Influence of the PPARα antagonist GW6471 on CBG-induced LC3-I to LC3-II conversion in A549 (A) and H460 cells (B). Figure S8. Effect of CBC (10 µM) on the release of mitochondrial cytochrome c (Cyt c) into the cytosol of A549 cells. Figure S9. Influence of the PPARα antagonist GW6471 on CBG-induced changes in OCR values in A549 (A,C) and H460 cells (B,D). Figure S10. Concentration-dependent effect of CBC on the concentrations of subunits of mitochondrial respiratory chain complexes in A549 (A,B) and H460 cells (C,D).
