# Supplementary material for: Cannabigerol and Cannabichromene Induce Lung Cancer Cell Death and Apoptosis—Contribution of PPARα to Cannabigerol Effects
Source: Antioxidants (Basel). 2026 Jun 15;15(6):754. doi: 10.3390/antiox15060754 (PMC13296359; doi:10.3390/antiox15060754)
Supplement: Supplementary file 1 [file antioxidants-15-00754-s001.zip › antioxidants-4078631-supplementary.pdf]

antioxidants

Supplementary Materials

Cannabigerol and Cannabichromene Induce Lung Cancer Cell Death and Apoptosis—Contribution of PPARα to Cannabigerol Effects

Theresa Spengler [1], Felix Wittig [1], Marcus Frank [2,3] and Burkhard Hinz [1,*]

[1] Institute of Pharmacology and Toxicology, Rostock University Medical Center, 18057 Rostock, Germany
[2] Electron Microscopy Center, Rostock University Medical Center, 18057 Rostock, Germany
[3] Department Life, Light and Matter, University of Rostock, 18059 Rostock, Germany
* Correspondence: burkhard.hinz@med.uni-rostock.de

 https://doi.org/10.3390/antiox15060754

Table S1. Concentration-dependent effects of CBG and CBC on survival and metabolic activity in A549 and H460 cells incubated with CBG or CBC for different periods of time. Survival rate and metabolic activity were assessed using the crystal violet (CV) assay and MTT assay, respectively. The IC_{50} values were determined based on the values shown in Figure 1 and represent the mean ± SEM of 4 biological replicates, each performed in technical triplicate.

Time (h)	CBG, IC_{50} (μM), Mean ± SEM				CBC, IC_{50} (μM), Mean ± SEM			
	A549		H460		A549		H460	
	CV	MTT	CV	MTT	CV	MTT	CV	MTT
2	n.c.	n.c.	n.c.	n.c.	n.c.	n.c.	n.c.	n.c.
6	8.00 ± 0.50	7.89 ± 0.43	9.98 ± 0.29	8.98 ± 0.31	16.68 ± 3.79	14.43 ± 2.66	36.60 ± 66.61	15.08 ± 8.09
24	6.25 ± 0.22	6.22 ± 0.21	4.94 ± 0.16	4.90 ± 0.29	9.84 ± 0.51	10.96 ± 1.06	8.01 ± 0.33	9.06 ± 0.99
48	5.65 ± 0.27	5.90 ± 0.41	4.40 ± 0.22	3.79 ± 0.27	10.15 ± 0.72	9.43 ± 0.62	7.39 ± 0.37	7.31 ± 0.81

n.c.: not calculable

https://doi.org/10.3390/antiox15060754

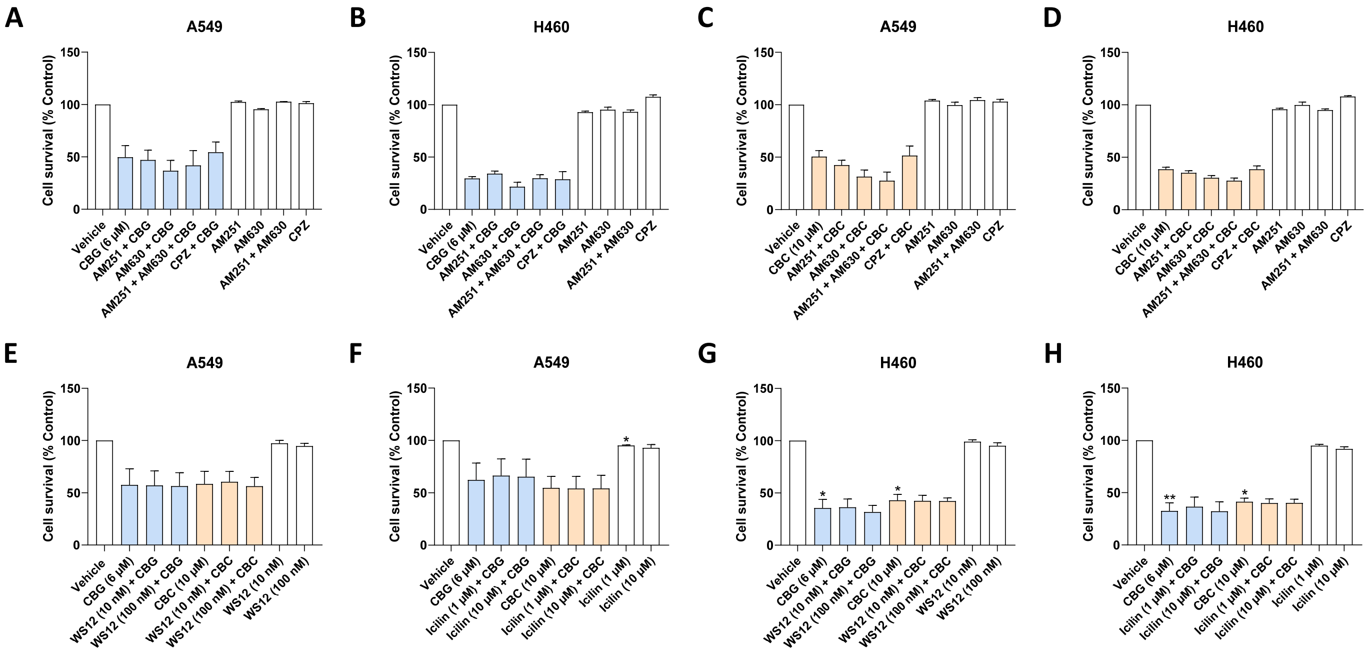

Supplementary Figure S1. Influence of antagonists of CB$_1$ (AM251), CB$_2$ (AM630), and TRPV1 (capsazepine, CPZ) (**A**–**D**), as well as agonists of TRPM8 (WS12, icilin) (**E**–**H**), on the decrease in cell survival mediated by CBG or CBC in A549 and H460 cells. Cells were pre-treated with receptor antagonists (each at 1 µM) or TRPM8 agonists at the indicated concentrations or their vehicle for 1 h, followed by a 24 h co-incubation with CBG (6 µM) or CBC (10 µM) or their vehicle. All percentage values shown refer to the respective time-matched vehicle control, which was set to 100%. Data represent the mean ± SEM of 3 (**A**–**D**) or 4 (**E**–**H**) biological replicates, each performed in technical triplicate. * $p \leq 0.05$, ** $p \leq 0.01$ vs. corresponding vehicle control; statistical analyses were performed on blank-corrected absorbance data using RM one-way ANOVA with Bonferroni's post hoc test (pre-specified comparisons).

https://doi.org/10.3390/antiox15060754

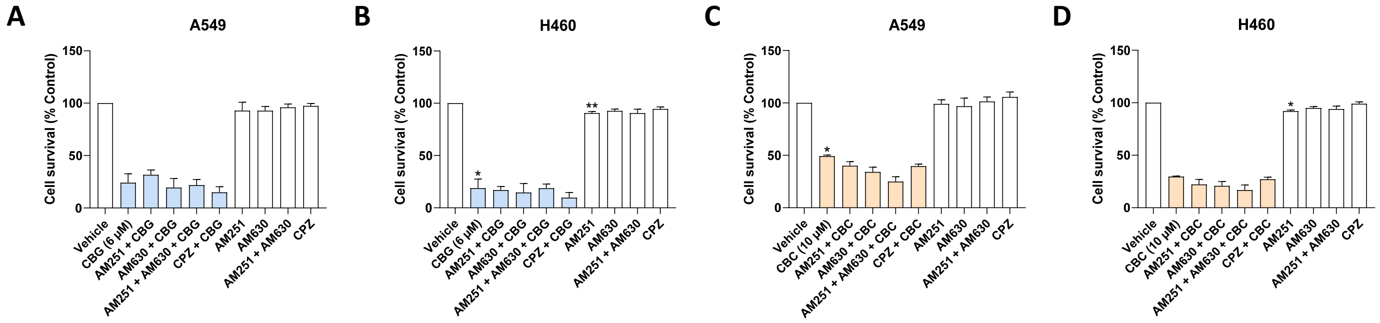

Supplementary Figure S2. Influence of antagonists of CB_1 (AM251), CB_2 (AM630), and TRPV1 (capsazepine, CPZ), each at 3 µM, on the decrease in cell survival mediated by CBG (**A**,**B**) or CBC (**C**,**D**) in A549 and H460 cells. Cells were pre-treated with receptor antagonists (each at 3 µM) or their vehicle for 1 h, followed by a 24 h co-incubation with CBG (6 µM) or CBC (10 µM) or their vehicle. All percentage values shown refer to the respective time-matched vehicle control, which was set to 100%. Data represent the mean ± SEM of 3 biological replicates, each performed in technical triplicate. * $p \leq 0.05$, ** $p \leq 0.01$ vs. corresponding vehicle control; statistical analyses were performed on blank-corrected absorbance data using RM one-way ANOVA with Bonferroni's post hoc test (pre-specified comparisons).

https://doi.org/10.3390/antiox15060754

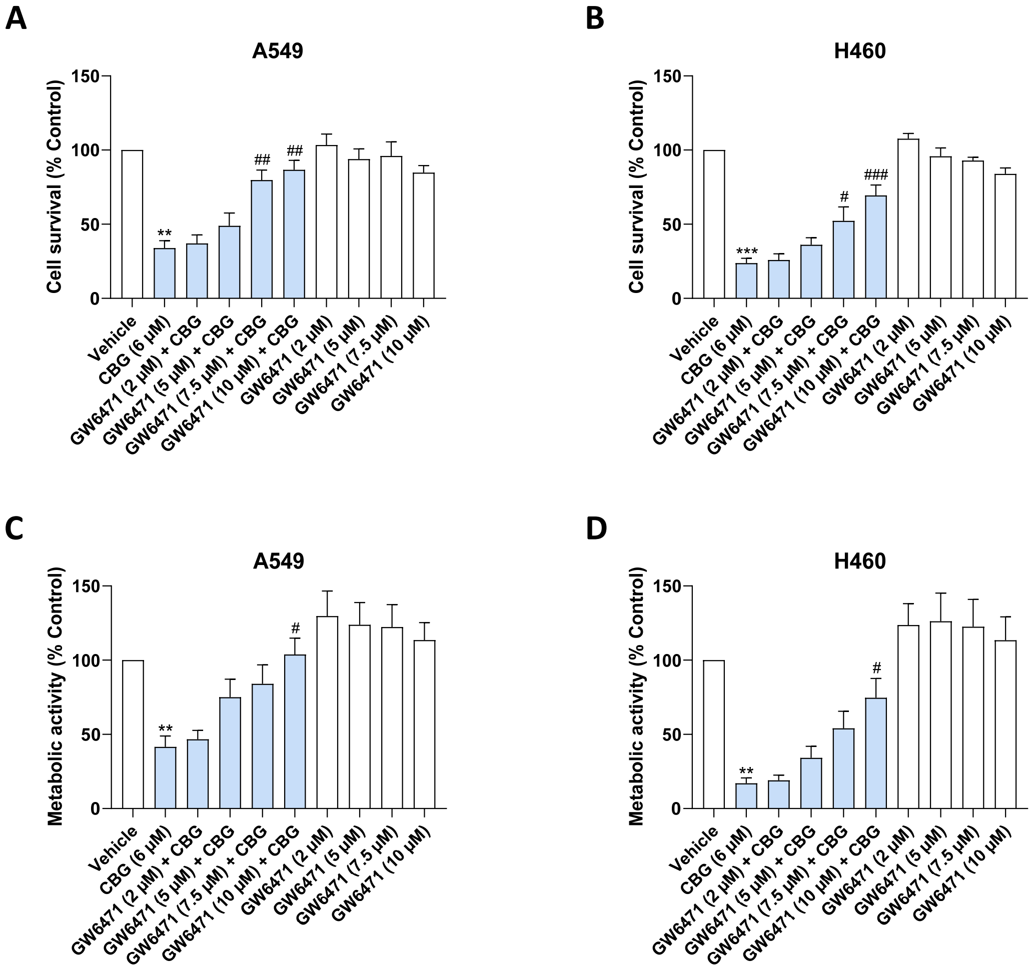

Supplementary Figure S3. Influence of the PPARα antagonist GW6471 on the decrease in cell survival (**A**,**B**) and metabolic activity (**C**,**D**) mediated by CBG in A549 and H460 cells. Cells were pretreated with GW6471 at the indicated concentrations or its vehicle for 1 h, followed by a 24 h co-incubation with CBG (6 µM) or its vehicle. Data represent the mean ± SEM of 8 biological replicates, each performed in technical triplicate. ** $p \leq 0.01$, *** $p \leq 0.001$ vs. corresponding vehicle control; # $p \leq 0.05$, ## $p \leq 0.01$, ### $p \leq 0.001$ vs. corresponding CBG-treated group; statistical analyses were performed on blank-corrected absorbance data using RM one-way ANOVA with Bonferroni's post hoc test (pre-specified comparisons).

https://doi.org/10.3390/antiox15060754

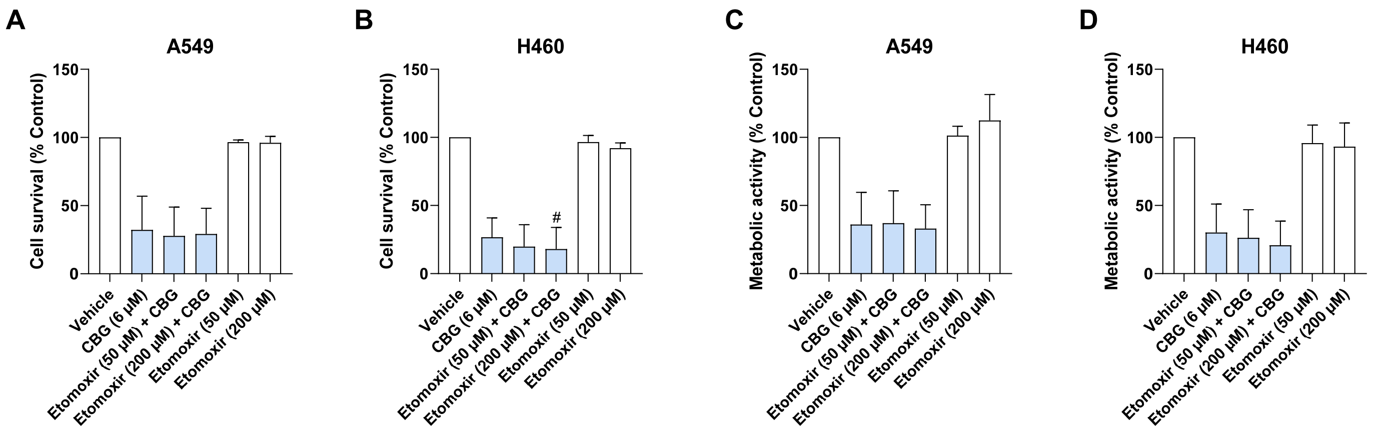

Supplementary Figure S4. Influence of the CPT1A antagonist etomoxir on the decrease in cell survival (**A**,**B**) and metabolic activity (**C**,**D**) mediated by CBG in A549 and H460 cells. Cells were pretreated with etomoxir at the indicated concentrations or its vehicle for 1 h, followed by a 24 h co-incubation with CBG (6 µM) or its vehicle. All percentage values shown refer to the respective time-matched vehicle control, which was set to 100%. Data represent the mean ± SEM of 3 biological replicates, each performed in technical triplicate. # $p \leq 0.05$ vs. corresponding CBG-treated group; statistical analyses were performed on blank-corrected absorbance data using RM one-way ANOVA with Bonferroni's post hoc test (pre-specified comparisons).

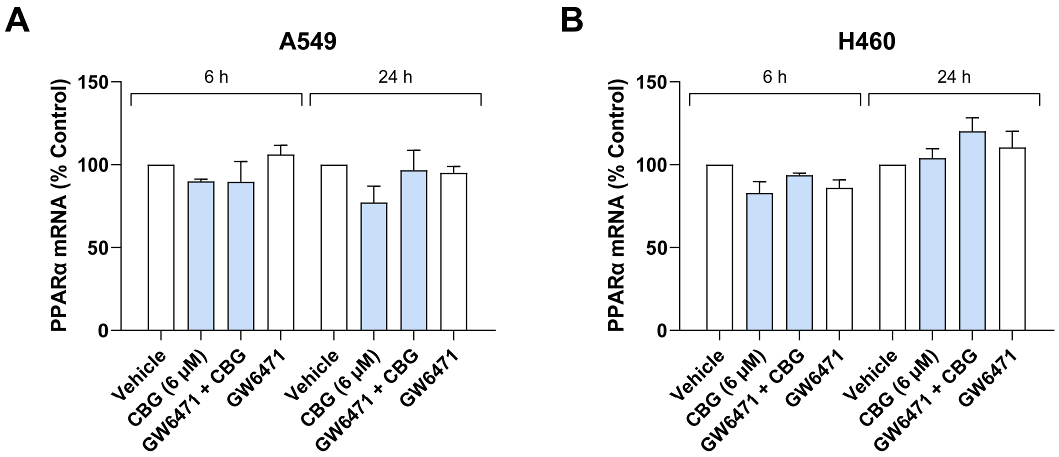

Supplementary Figure S5. Effect of CBG and the PPARα antagonist GW6471, tested alone or in combination, on PPARα mRNA expression in A549 (**A**) and H460 cells (**B**). Cells were pre-treated with GW6471 (10 μM) or its vehicle for 1 h, followed by co-incubation with CBG (6 μM) or its vehicle for 6 or 24 h. PPARα mRNA levels were measured by qRT-PCR and normalized to PPIA mRNA. All percentages shown refer to the respective vehicle control (100%). Data represent the mean ± SEM of 3 biological replicates. Statistical analyses were performed on ΔCt values using RM one-way ANOVA with Bonferroni's post hoc test (pre-specified comparisons). No significant differences between groups were observed.

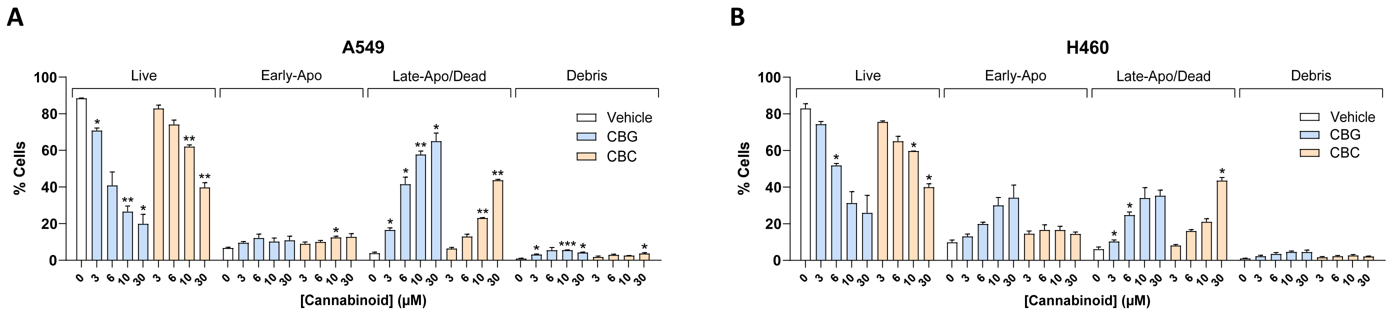

Supplementary Figure S6. Concentration-dependent effects of CBG and CBC (6 h treatment) on early and late apoptosis in A549 (**A**) and H460 cells (**B**), as determined by the Muse® Annexin V & Dead Cell Kit. Cells were incubated for 6 h with the respective cannabinoid at the specified concentrations or its vehicle. The cells were then stained using the Muse® Annexin V & Dead Cell Kit to distinguish between living (Live), early apoptotic (Early-Apo), late apoptotic/dead (Late-Apo/Dead), and non-apoptotic debris. Data represent the mean ± SEM of 3 biological replicates. * $p \leq 0.05$, ** $p \leq 0.01$, *** $p \leq 0.001$ vs. corresponding vehicle control; statistical analyses were performed on percentages of gated cells using RM one-way ANOVA with Dunnett´s post hoc test.

https://doi.org/10.3390/antiox15060754

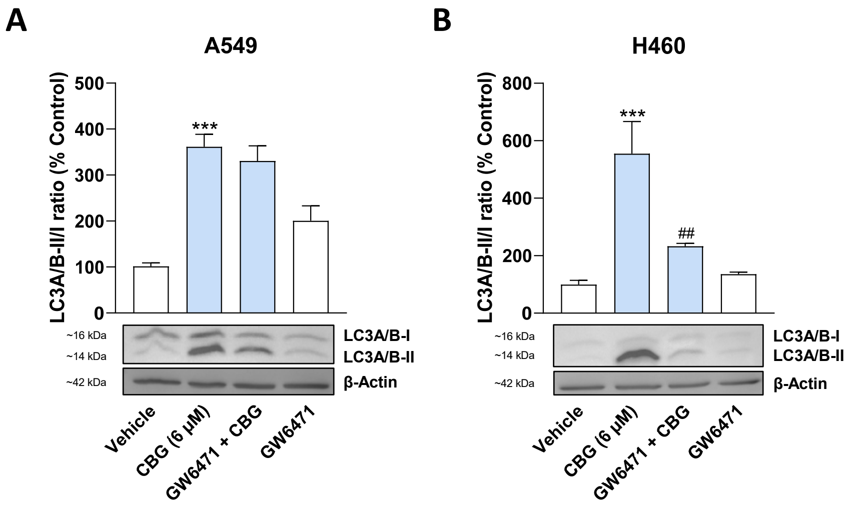

Supplementary Figure S7. Influence of the PPARα antagonist GW6471 on CBG-induced LC3-I to LC3-II conversion in A549 (**A**) and H460 cells (**B**). Cells were pre-treated with GW6471 (10 µM) or its vehicle for 1 h, followed by a 24 h co-incubation with CBG (6 µM) or its vehicle. Bar chart values were derived from densitometric analyses of the blots. All percentages refer to the respective vehicle control (mean = 100%). The blots shown are representative. Data represent the mean ± SEM of $n = 4$ biological replicates. *** $p \leq 0.001$ vs. corresponding vehicle control; ## $p \leq 0.01$ vs. corresponding CBG-treated group; statistical analyses were performed on normalized data expressed as percentages of the respective vehicle control using one-way ANOVA with Bonferroni's post hoc test (pre-specified comparisons).

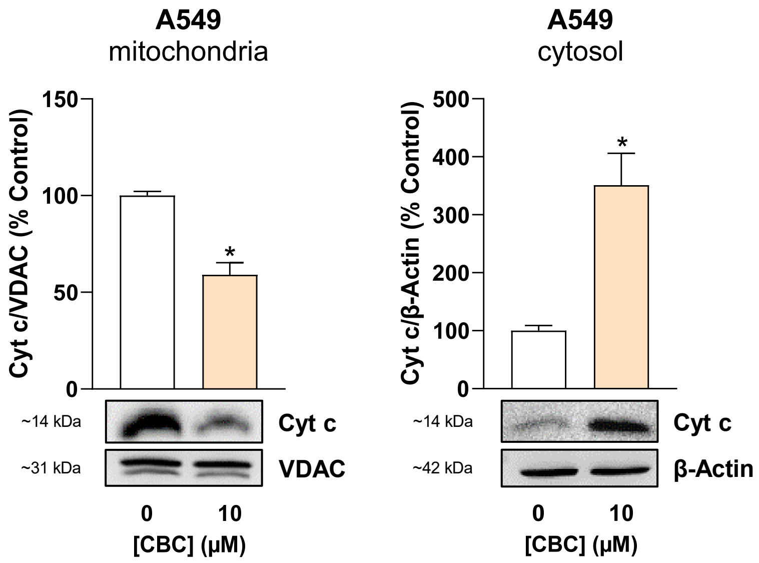

Supplementary Figure S8. Effect of CBC (10 μM) on the release of mitochondrial cytochrome c (Cyt c) into the cytosol of A549 cells. Cells were incubated with CBC or its vehicle for 2 h. Bar chart values were derived from densitometric analyses of the blots. Cyt c levels were normalized to VDAC in mitochondrial fractions and to β-actin in cytosolic fractions. All percentages refer to the respective vehicle control (mean = 100%). The blots shown are representative. Data represent the mean ± SEM of 3 biological replicates. * $p \leq 0.05$ vs. vehicle control; statistical analyses were performed on VDAC- and β-actin-normalized values expressed as percentages of the respective vehicle control using unpaired two-tailed Welch's t-test.

https://doi.org/10.3390/antiox15060754

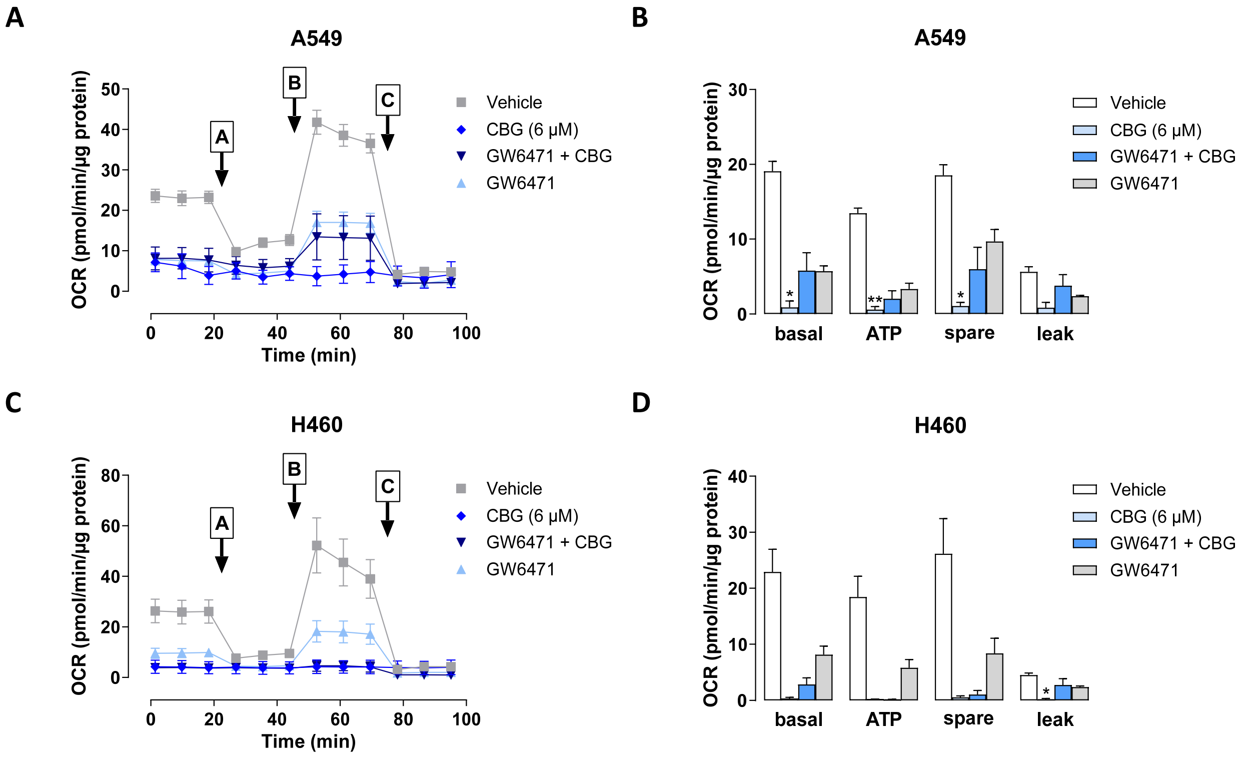

Supplementary Figure S9. Influence of the PPARα antagonist GW6471 on CBG-induced changes in OCR values in A549 (**A**,**B**) and H460 cells (**C**,**D**). Cells were pre-treated with GW6471 (10 µM) or its vehicle for 1 h, followed by a 24 h co-incubation with CBG (6 µM) or its vehicle. A mitochondrial stress test was then carried out and OCR values were determined using the Seahorse XFe24 Analyzer. Therefore, oligomycin (port A), FCCP (port B) and antimycin A/rotenone (port C) were loaded into the respective ports of the sensor cartridges and released into the wells at the specified times. From this assay, the time courses of OCR (**A**,**C**) in both cell lines treated with CBG and/or GW6471 are shown, as well as calculations (**B**,**D**) of basal respiration (basal), ATP-linked respiration (ATP), spare respiratory capacity (spare) and proton leak. Data represent the mean ± SEM of 3 biological replicates, performed in duplicates to quadruplicates. * $p \leq 0.05$, ** $p \leq 0.01$ vs. corresponding vehicle control; statistical analyses were performed on protein-normalized OCR values using RM one-way ANOVA with Bonferroni´s post hoc test (pre-specified comparisons).

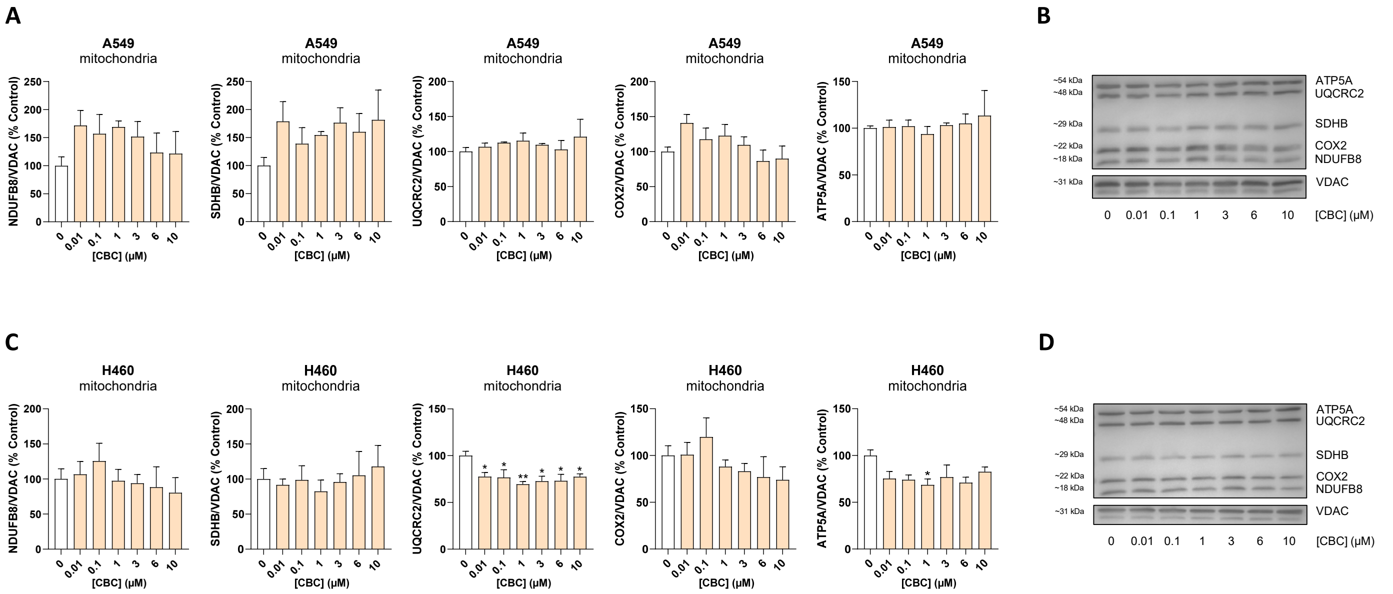

Supplementary Figure S10. Concentration-dependent effect of CBC on the concentrations of subunits of mitochondrial respiratory chain complexes in A549 (**A**,**B**) and H460 cells (**C**,**D**). Cells were treated with the indicated concentrations of CBC for 24 h. Thereafter, the corresponding proteins in the mitochondrial fractions were determined using Western blot analysis. Bar chart values were derived from densitometric analyses of the blots. Mitochondrial proteins were normalized to VDAC. All percentages refer to the respective vehicle control (mean = 100%). The blots shown are representative. In (**D**) the same VDAC blot is shown as in Figure 13D, as the same membranes were stripped and reprobed for different target proteins. Data represent the mean ± SEM of 3 biological replicates. * $p \leq 0.05$, ** $p \leq 0.01$ vs. corresponding vehicle control; statistical analyses were performed on VDAC-normalized data expressed as percentages of the respective vehicle control using one-way ANOVA with Dunnett's post hoc test.

Disclaimer/Publisher's Note: The statements, opinions and data contained in all publications are solely those of the individual author(s) and contributor(s) and not of MDPI and/or the editor(s). MDPI and/or the editor(s) disclaim responsibility for any injury to people or property resulting from any ideas, methods, instructions or products referred to in the content.

https://doi.org/10.3390/antiox15060754
